# Improved CKD classification based on explainable artificial intelligence with extra trees and BBFS

**DOI:** 10.1038/s41598-025-02355-7

**Published:** 2025-05-22

**Authors:** Ahmed M. Elshewey, Enas Selem, Amira Hassan Abed

**Affiliations:** 1 Department of Computer Science, Faculty of Computers and Information, Suez University, P.O.BOX:43221, Suez, Egypt; 2 Department of Information Technology, Faculty of Computers and Information, Suez University, P.O.BOX:43221, Suez, Egypt; 3 Department of Information Systems, High Institution for Marketing, Commerce & Information Systems, Cairo, Egypt

**Keywords:** Chronic kidney disease, Explainable artificial intelligence, Extra trees classifier, SHAP, Binary breadth-first search, Feature selection, Kidney diseases, Computer science, Information technology

## Abstract

Chronic kidney disease is a persistent ailment marked by the gradual decline of kidney function. Its classification primarily relies on the estimated glomerular filtration rate and the existence of kidney damage. The kidney disease improving global outcomes organization has established a widely accepted system for categorizing chronic kidney disease. explainable artificial intelligence for classification involves creating machine learning models that not only accurately predict outcomes but also offer clear and interpretable explanations for their decisions. Traditional machine learning models often pose difficulties in comprehending the intricate processes behind specific classification choices due to their intricate and obscure nature. In this study, an explainable artificial intelligence-chronic kidney disease model is introduced for the process of classification. The model applies explainable artificial intelligence by utilizing extra trees and shapley additive explanations values. Also, binary breadth-first search algorithm is used to select the most important features for the proposed explainable artificial intelligence-chronic kidney disease model. This methodology is designed to derive valuable insights for enhancing decision-making strategies within the field of classifying chronic kidney diseases. The performance of the proposed model is compared with another machine learning models, namely, random forest, decision tree, bagging classifier, adaptive boosting, and k-nearest neighbor, and the performance of the models is evaluated using accuracy, sensitivity, specificity, F-score, and area under the ROC curve. The experimental results demonstrated that the proposed model achieved the best results with accuracy equals 99.9%.

## Introduction

Chronic kidney disease (CKD) represents a pervasive and progressively debilitating health condition that demands early detection and accurate classification for effective intervention and management^1^. It is diagnosed by a glomerular filtration rate (GFR) persistently below a certain threshold for over three months or the presence of indicators of kidney damage. Common contributors to CKD encompass diabetes, hypertension, glomerulonephritis, and related conditions. With a global impact affecting millions, CKD imposes a substantial healthcare burden due to its widespread prevalence, costly treatments, and potential life-threatening complications. Timely detection and identification of key risk factors are pivotal in effectively managing CKD and enhancing patient outcomes. Conventional diagnostic approaches have proven inadequate in accurately predicting CKD progression and discerning the most influential factors contributing to its onset. In recent years, the convergence of advanced machine learning techniques and medical research has opened new avenues for improving the precision and interpretability of CKD classification models^2,3^. Chronic diseases are types of non-communicable illness and are a major cause of declining physical health and mental well-being. They are among the top causes of illness and death worldwide. While these conditions can be complex, many are preventable if identified and addressed early. To support better clinical decision-making, predictive models have been developed to help doctors and patients. These models analyze various risk factors to assess an individual’s likelihood of developing a chronic disease, providing a clearer path for early intervention and prevention^4^. This convergence signifies a significant leap forward in addressing the complexities of chronic kidney disease (CKD) classification. Advanced machine learning techniques, fueled by the latest developments in artificial intelligence, are now intricately woven into the fabric of medical research. This integration has ushered in a new era where the precision and interpretability of CKD classification models can be finely tuned, offering a more nuanced and comprehensive understanding of the disease. In the realm of CKD, where early detection and effective risk assessment are paramount, the synergy of advanced machine learning techniques and medical research facilitates a more granular exploration of the intricate patterns and indicators associated with the disease. The precision of these models is enhanced, allowing for a more accurate identification of CKD stages and potential risk factors. Moreover, the focus on interpretability brings transparency to the decision-making processes of these models. This transparency is crucial in gaining the trust of healthcare professionals and end-users who require insights into how predictions are made. The ability to interpret and explain the model’s reasoning empowers clinicians to make informed decisions and fosters greater acceptance of these advanced techniques in the medical community. Artificial intelligence (AI), machine learning (ML), and deep learning have advanced rapidly, bringing significant improvements to industries like healthcare. The abundance of medical diagnostic data has made it possible to train powerful algorithms that drive innovation and enhance outcomes^5–10^. As researchers delve deeper into understanding the multifaceted nature of CKD, the marriage of advanced machine learning and medical research not only refines the accuracy of predictions but also opens avenues for discovering novel biomarkers and subtle nuances that may have previously eluded traditional diagnostic approaches. The majority of machine learning (ML) models are often characterized as “black boxes,” referring to models that are complex enough to defy easy interpretation by humans^11,12^. When employing such black-box models as diagnostic tools, the challenge arises for healthcare professionals to comprehend the factors that led the model to make a specific prediction^13^. This lack of transparency in black-box approaches poses obstacles to medical decision support from both physicians’ and patients’ perspectives. Therefore, there is a pressing need to develop diagnostic systems that offer interpretability for ML models. The interpretability of ML models serves as a crucial aspect for creating a diagnostic system that enables physicians to understand and trust the results provided^14^. This transparency acts as a safety check on predicted outcomes, fostering confidence among physicians in the system’s decision-making process. Consequently, the quest for interpretability has fueled growing interest in the field of explainable artificial intelligence (XAI). Explainable ML models, also referred to as interpretable ML models, belong to the field of explainable artificial intelligence (XAI), defined by Arrieta et al.^15^ as follows: “An explainable Artificial Intelligence is one that provides details or reasons to make its functioning clear or easy to understand for a given audience.” Consequently, XAI enables healthcare experts to make informed, data-driven decisions, facilitating the delivery of more personalized and reliable treatments^16^. However, a fundamental tension arises between the predictive accuracy and interpretability of ML models, as the best-performing models often lack transparency, while those with clear explanations (e.g., decision trees) may sacrifice accuracy. In the healthcare domain, the limited explainability hinders the broader adoption of ML solutions, as healthcare professionals may find it challenging to trust complex models that require high technical and statistical knowledge. In this paper, we propose an innovative approach that leverages Explainable artificial intelligence (XAI) in conjunction with extra trees, an ensemble learning algorithm, to enhance the classification accuracy and transparency of CKD diagnostic systems. The importance of precise CKD classification appears from the necessity to recognize and interpose in the very early stages of the disease, permitting healthcare providers to tailor treatments and interventions to the certain needs of each patient. The integration of machine learning techniques offers a promising avenue to achieve this goal, with the added advantage of providing transparent insights into the decision-making process of the model. Our approach focuses on the exploitation of Extra Trees, an ensemble learning method famous for its capability to handle complex datasets and provide robust predictions^17^. By incorporating a degree of randomness during the construction of decision trees^18^, Extra Trees enhances diversity among the individual trees, potentially improving the model’s overall performance. However, the intrinsic complexity of such models often raises concerns about interpretability. To address this interpretability challenge, we introduce XAI techniques to our CKD classification model. XAI aims to demystify the decision-making process of machine learning models, offering insights into the factors influencing individual predictions. By employing methods such as local interpretable model-agnostic explanations (LIME)^19^ or shapley additive explanations (SHAP)^20^, we strive to make our CKD classification model not only accurate but also transparent and understandable to healthcare professionals and patients. In the subsequent sections of this paper, we delve into the methodology employed in integrating Extra Trees and XAI into the CKD classification process. We explore the significance of feature importance analysis, discuss the interpretability of the model through XAI techniques, and present experimental results validating the efficacy of our proposed approach. By fusing the power of Extra Trees with the interpretability of XAI, our research contributes to the ongoing efforts in creating advanced, transparent, and clinically relevant models for CKD classification. The implications of this work extend beyond the realm of kidney disease, providing a blueprint for the development of explainable and accurate diagnostic systems in the broader landscape of medical artificial intelligence.

Despite the advances in chronic kidney disease classification using machine learning, there remains a significant gap in balancing model performance with interpretability. Many high-performance models are considered “black boxes,” limiting their adoption in clinical settings due to a lack of transparency. Furthermore, feature selection methods in existing studies often rely on metaheuristics and statistical filters that may not efficiently capture dependencies in high-dimensional medical data. To address this, we identified the need for a robust and interpretable solution that also efficiently selects the most relevant features. This forms the basis of our proposed approach, which combines binary breadth-first search (BBFS) for feature selection with extra trees ensemble model and shapley additive explanations (SHAP) for interpretability.

The main contribution in this paper is using an explainable artificial intelligence-CKD (XAI-CKD) model for the process of classification. XAI-CKD applies explainable AI (XAI) by utilizing extra trees (ET) and SHAP (shapley additive explanations) values. Also, binary breadth-first search (BBFS) algorithm is used to select the most important features for XAI-CKD model. BBFS is an efficient feature selection algorithm, making it particularly well-suited for CKD datasets, which often have high-dimensional features and interdependencies. BBFS works effectively via traversing the feature space to identify the most relevant features without requiring an exhaustive search, this saves computational resources. Extra Trees is an ensemble learning model used for capturing complex relationships between features through randomized tree construction. By combining BBFS with ET, noise in the dataset is reduced via selecting only the most important features, this helps in improving model performance and interpretability. This integration makes a balance between computational efficiency, accuracy, and clarity the results, which is vital in a medical context like CKD classification. This methodology is designed to derive valuable insights for enhancing decision-making strategies within the field of classifying chronic kidney diseases. The performance of XAI-CKD is compared with another machine learning models, namely, random forest (RF), decision tree (DT), bagging classifier (BC), adaptive boosting (AdaBoost), and k-nearest neighbor (KNN), and the performance of the models is evaluated using accuracy, sensitivity, specificity, F-score, and area under the ROC curve (AUC). The proposed XAI-CKD model achieved the best results with accuracy equals 99.9%. The steps for creating XAI-CKD model for chronic kidney disease classification are:

- Data collection.
- Data normalization using Z-score.
- Using KNN imputer for imputing missing values.
- Using binary breadth-first search (BBFS) algorithm to identify the most relevant features for the classification task.
- Select the extra trees (ET) classification model that is inherently explainable and can be easily interpreted.
- Train the model by splitting the data into training (70%), and testing (30%) sets.
- Evaluate model performance using evaluation metrics such as accuracy, sensitivity, specificity, F-score, and area under the ROC curve (AUC).
- Generate explanations for the model predictions. SHAP (shapley additive explanations) is used, which provides insights into how the model arrives at specific predictions.

The rest of this paper is organized as follows: Sect. 2 presents related work on CKD classification. Section 3 describes the materials and methods. Section 4 discusses experimental results. Section 5 outlines the limitations of the current study. Section 6 concludes the paper and suggests future research directions. Section 7 addresses ethical considerations in AI-based CKD classification.

## Related work

In recent years, machine learning approaches plays an important role in the medical field^21–25^. The application of machine learning (ML) models for predicting chronic kidney diseases (CKD) has garnered significant attention. However, the need for transparency and interpretability in these models has led to a surge in research focusing on explainable machine learning (XML) approaches within the realm of CKD prediction. Here is an overview of machine learning methods that support chronic kidney disease (CDK) prediction. Traditional ML Approaches: Early efforts in CKD prediction primarily involved conventional ML techniques, such as logistic regression^26^, decision trees^27^, support vector machines (SVM) in CKD Prediction^28^ and Naive Bayes in CKD Diagnosis^29^. While these models exhibited reasonable performance, their interpretability was limited, hindering their adoption in clinical practice. Therefore, Interpretability Challenges in Black-Box Models have been presented. The advent of sophisticated black-box models, including deep neural networks and ensemble methods^30^, marked a significant shift towards improved predictive accuracy. However, the opacity of these models posed challenges in understanding the rationale behind predictions, particularly in the context of CKD. For this reason, explainable artificial intelligence (XAI) appeared to cover this problem. explainable artificial intelligence (XAI): Recognizing the need to balance predictive accuracy with interpretability, the research community embraced explainable artificial intelligence (XAI). XAI techniques, such as rule-based models^28^, feature importance analysis^29^, and shapley additive explanations (SHAP)^30^, emerged as pivotal tools for unraveling the decision-making processes of complex ML models. Now we will explore recent work, the exploit Explainable Artificial Intelligence in CKD Prediction. In^31^ Two ensemble approaches, namely XGBoost and AdaBoost, have been employed for the detection of chronic kidney disease (CKD). Subsequently, a model leveraging deep neural networks (DNN) is proposed for CKD prognosis, wherein hyperparameters were fine-tuned to enhance performance. Impressively, the DNN outperformed the ensemble methods, achieving an accuracy of 98.8%. To shed light on feature contributions and enhance interpretability, the study incorporated shapley additive explanations (SHAP), a technique gaining prominence in explainable artificial intelligence (XAI). The SHAP analysis aimed to discern the features influencing CKD prediction, confirming alignment with medical explanations. In^20^ the proposed system, rooted in the decision tree-based explainable artificial intelligence (DT-EAI), aims to furnish a robust solution for early diagnosis. Employing a Data-driven approach, the system leverages the chronic kidney disease (CKD) dataset, conducting feature selection based on Gini Importance values. Subsequently, it constructs a model utilizing the Decision Tree algorithm and interprets the model using SHAP values. To ensure robustness, the system evaluates and validates its performance through Cross-Validation, undergoing iterative refinement based on feedback from healthcare professionals. This iterative process hones the model for enhanced accuracy and interpretability. Upon achieving satisfactory performance, the system is deployed for practical use in early diagnosis. The system’s effectiveness is gauged using evaluation metrics such as the F1 score and fidelity accuracy index (FAI). In^32^, the classification of chronic kidney disease (CKD) was carried out using the XGBoost classifier. To optimize the feature set, binary bat optimization (BBO) was employed to reduce the number of features and obtain an optimal subset. The model exhibited remarkable performance with all 24 features, yielding an accuracy, precision, recall, and F1 score of 99.16%, 100%, 98.68%, and 99.33%, respectively. Moreover, utilizing only the 13 features selected by the BBO algorithm maintained strong metrics, achieving an accuracy, precision, recall, and F1 score of 98.33%, 100%, 97.36%, and 98.67%, respectively. The analysis of machine learning models trained on both the original set and the BBO-selected feature subset using shapley additive explanations (SHAP) revealed that hemoglobin and albumin exerted significant influence on the model. Interestingly, the BBO algorithm also identified these attributes, along with a few additional traits, as the most impactful features. The study demonstrated the nuanced impact of each feature on the model’s classification of a single sample within a specific class, offering valuable insights for clinicians. The transparency provided by explaining the black-box machine learning model proves advantageous for both clinicians and patients. The proposed system, implementable in any hospital, stands to support less experienced nephrologists in achieving more accurate CKD diagnoses. The authors suggest that future research could explore even more sophisticated explainable artificial intelligence (XAI) methods to further enhance the interpretability of the machine learning model. In^33^ introduces the development and assessment of an explainable chronic kidney disease (CKD) prediction model designed to elucidate how various clinical features contribute to the early diagnosis of CKD. The model is crafted within an optimization framework that seeks to strike a balance between classification accuracy and interpretability. The primary contribution of this research lies in its embrace of an explainable data-driven methodology, providing quantifiable insights into the role of specific clinical features in the early detection of CKD. The optimal explainable prediction model is constructed using an extreme gradient boosting classifier, focusing on three key features: hemoglobin, specific gravity, and hypertension. This model achieves an accuracy of 99.2% (standard deviation 0.8) and 97.5% with a 5-fold cross-validation and new, unseen data, respectively. An insightful explain ability analysis underscores the significance of hemoglobin as the most influential feature shaping predictions, closely followed by specific gravity and hypertension. The utilization of this minimal set of features not only enhances the accuracy of early CKD diagnosis but also presents a cost-effective solution, particularly relevant for developing countries. The study in^34^ focuses on developing a classifier model aimed at assisting healthcare professionals in the early diagnosis of CKD patients. Employing a comprehensive data pipeline, the researchers conduct an exhaustive search to identify the optimal data mining classifier, considering various parameters within the data preparation stages such as handling missing data and feature selection. Subsequently, Extra Trees emerges as the most effective classifier, achieving remarkable accuracies of 100% with cross-validation and 99% with new, unseen data. Furthermore, the study utilizes the eight selected features to evaluate the explainability of the model’s outcomes, providing insights into the relative importance of each feature in influencing the model’s predictions. The study in^35^ explores the use of explainable artificial intelligence (XAI) techniques to predict chronic kidney disease (CKD) based on clinical characteristics. Clinical data from 491 patients, including 56 with CKD, were analyzed, encompassing various clinical, laboratory, and demographic variables. Five machine learning methods were employed, and the extreme gradient boosting (XGBoost) model emerged as the most effective, achieving an accuracy of 93.29% and an AUC of 0.9689. Feature importance analysis highlighted creatinine, glycosylated hemoglobin type A1C (HgbA1C), and age as the most influential features. SHAP and LIME algorithms were utilized for interpretability, providing insights into individual predictions and enhancing clinicians’ understanding of the model’s rationale. This approach presents an interpretable ML-based strategy for early CKD prediction, facilitating better-informed clinical decision-making. The work in^36^, an explainable machine learning model was constructed to forecast chronic kidney disease. The methodology involved building an automated data pipeline utilizing the Random Forest ensemble learning trees model alongside a feature selection algorithm. The model’s explainability was evaluated through assessments of feature importance and explainability metrics. Three distinct explainability methods: LIME, SHAP, and SKATER were employed to interpret the model’s outcomes. Furthermore, comparisons of explainability results were conducted using interpretability, fidelity, and fidelity-to-interpretability ratio as the key explainability metrics. The work in^37^ introduces a novel hybrid approach for diagnosing Chronic Renal Disease, which optimizes an SVM classifier through a hybridized dimensionality reduction technique. It employs a two-step feature selection process: first, a filter-based approach using ReliefF assigns weights and ranks to each feature, followed by dimensionality reduction using PCA. The model also utilizes simultaneous execution on multiple processors for faster processing. Results show that the proposed approach achieves significantly higher prediction accuracies compared to existing methods on both clinical CKD and benchmarked chronic kidney disease datasets.

## Materials and methods

In this study, an explainable artificial intelligence-CKD (XAI-CKD) model is introduced for the process of classification. XAI-CKD applies explainable AI (XAI) by utilizing extra trees (ET) and SHAP (shapley Additive explanations) values. Also, binary breadth-first search (BBFS) algorithm is used to select the most important features for XAI-CKD model. BBFS is an efficient feature selection algorithm, making it particularly well-suited for CKD datasets, which often have high-dimensional features and interdependencies. BBFS works effectively via traversing the feature space to identify the most relevant features without requiring an exhaustive search, this saves computational resources. Extra Trees is an ensemble learning model used for capturing complex relationships between features through randomized tree construction. According to no free lunch (NFL) theorem^38^, which demonstrates that no single classifier works best for every problem. Our study does not claim that extra trees are the best. However, in the specific context of our dataset and problem, focusing on CKD classification and the XAI-CKD model with extra trees provides balance of accuracy and interpretability. By combining BBFS with ET, noise in the dataset is reduced via selecting only the most important features, this helps in improving model performance and interpretability. This integration makes a balance between computational efficiency, accuracy, and clarity the results, which is vital in a medical context like CKD classification. This methodology is designed to derive valuable insights for enhancing decision-making strategies within the field of classifying chronic kidney diseases. The performance of XAI-CKD is compared with another machine learning models, namely, random forest (RF), decision tree (DT), bagging classifier (BC), adaptive boosting (AdaBoost), and k-nearest neighbor (KNN), and the performance of the models is evaluated using accuracy, sensitivity, specificity, F-score, and area under the ROC curve (AUC). The steps for creating XAI-CKD model for chronic kidney disease classification:

1. Data collection.
2. Data normalization using Z-score.
3. Using KNN imputer for imputing missing values.
4. Using binary breadth-first search (BBFS) algorithm to identify the most relevant features for the classification task.
5. Select the extra trees (ET) classification model that is inherently explainable and can be easily interpreted.
6. Train the model by **s**plitting the data into training (70%), and testing (30%) sets.
7. Evaluate model performance using evaluation metrics such as accuracy, sensitivity, specificity, F-score, and area under the ROC curve (AUC).
8. Generate explanations for the model predictions. SHAP (shapley additive explanations) is used, which provides insights into how the model arrives at specific predictions.

**Fig. 1:**
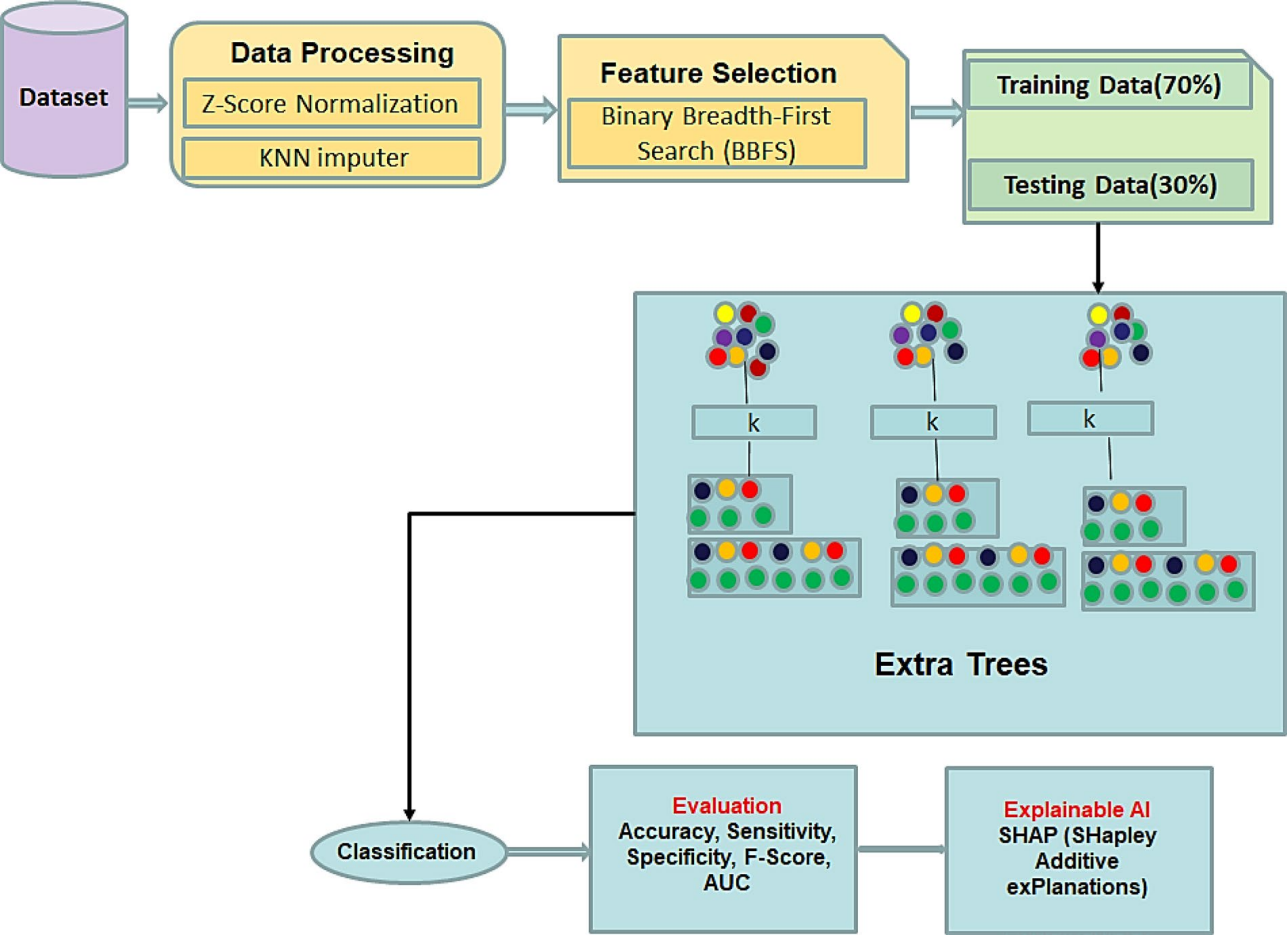
Framework of the proposed methodology.

The framework of the proposed methodology is depicted in Fig. 1.

### Dataset

**Fig. 2:**
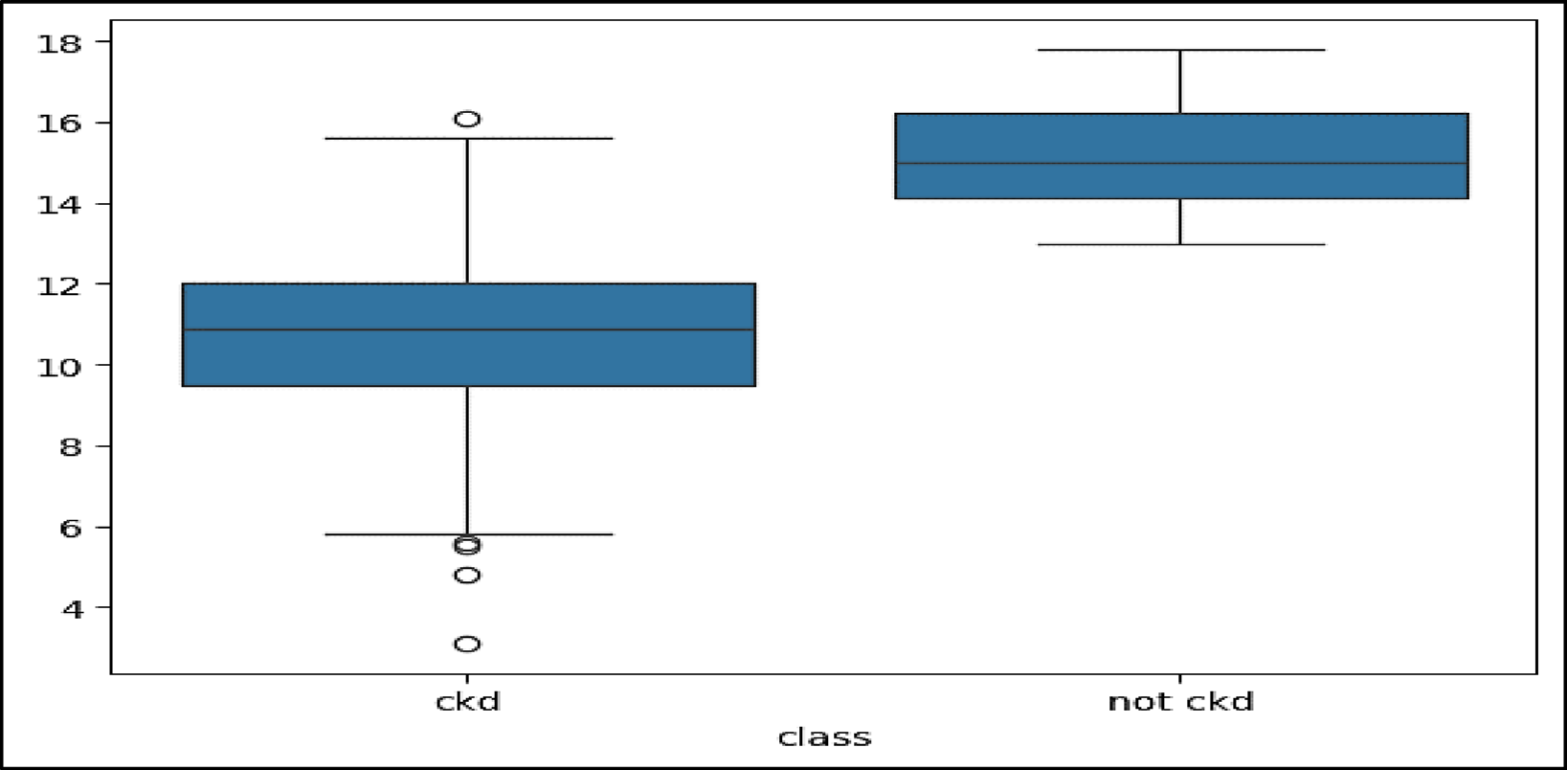
Boxplot visualization for the values of the target features.

The dataset used in this paper is available at^39^. The hospital in Karaikudi, Tamilnadu, India provided this data set. There are 25 features in the dataset, 400 patients are employed for training algorithms that use ML to do classification. As a result, the target feature is marked in two classes: CKD and not-CKD. The dataset features include: age, blood pressure, specific gravity, albumin, sugar, red blood cells, pus cell, pus cell clumps, bacteria, blood glucose random, blood urea, serum creatinine, sodium, potassium, hemoglobin, packed cell volume, white blood cell count, red blood cell count, hypertension, diabetes mellitus, coronary artery disease, appetite, Peda edema, anemia, and class (target label: CKD or not-CKD). To handle missing values, we applied k-nearest neighbors (KNN) imputer during preprocessing. Z-score normalization was then used to standardize the dataset. There are medically significant factors in these data records that may be linked to the existence of CKD. Figure 2 represents the boxplot visualization for the values of the target feature. Boxplot visualization is crucial lies in its ability to give a brief yet insightful summary of how data is spread out and its variability. Boxplots are valuable in analyzing data because they offer a quick snapshot of important statistical measures like the center, spread, and any outliers present^40^. They allow researchers to understand the fundamental characteristics of data distribution without delving into complex numerical analysis, making them extremely useful for exploring and visualizing data^41^.

Table 1 provides a summary of important statistical properties for each feature in the dataset:

1. Feature Name: This column lists the names of the features or variables in the dataset.
2. Count: It shows the number of observations or data points available for each feature. In this case, there are 400 recorded patients for each feature.
3. Mean: This indicates the average value of the feature across all observations. It’s calculated by adding up all values and then dividing them by the total number of observations.
4. Std (Standard deviation): This measures the spread or dispersion of the data around the mean. It tells us how much values deviate from the mean. A higher standard deviation suggests greater variability in the dataset.
5. Min (Minimum): It displays the smallest observed value for each feature in the dataset.
6. 50% (Median): This value represents the median of the feature. The median is the middle value when observations are sorted in ascending order, dividing the dataset into two equal halves.
7. Max (Maximum): This indicates the largest observed value for each feature in the dataset.

**Fig. 3:**
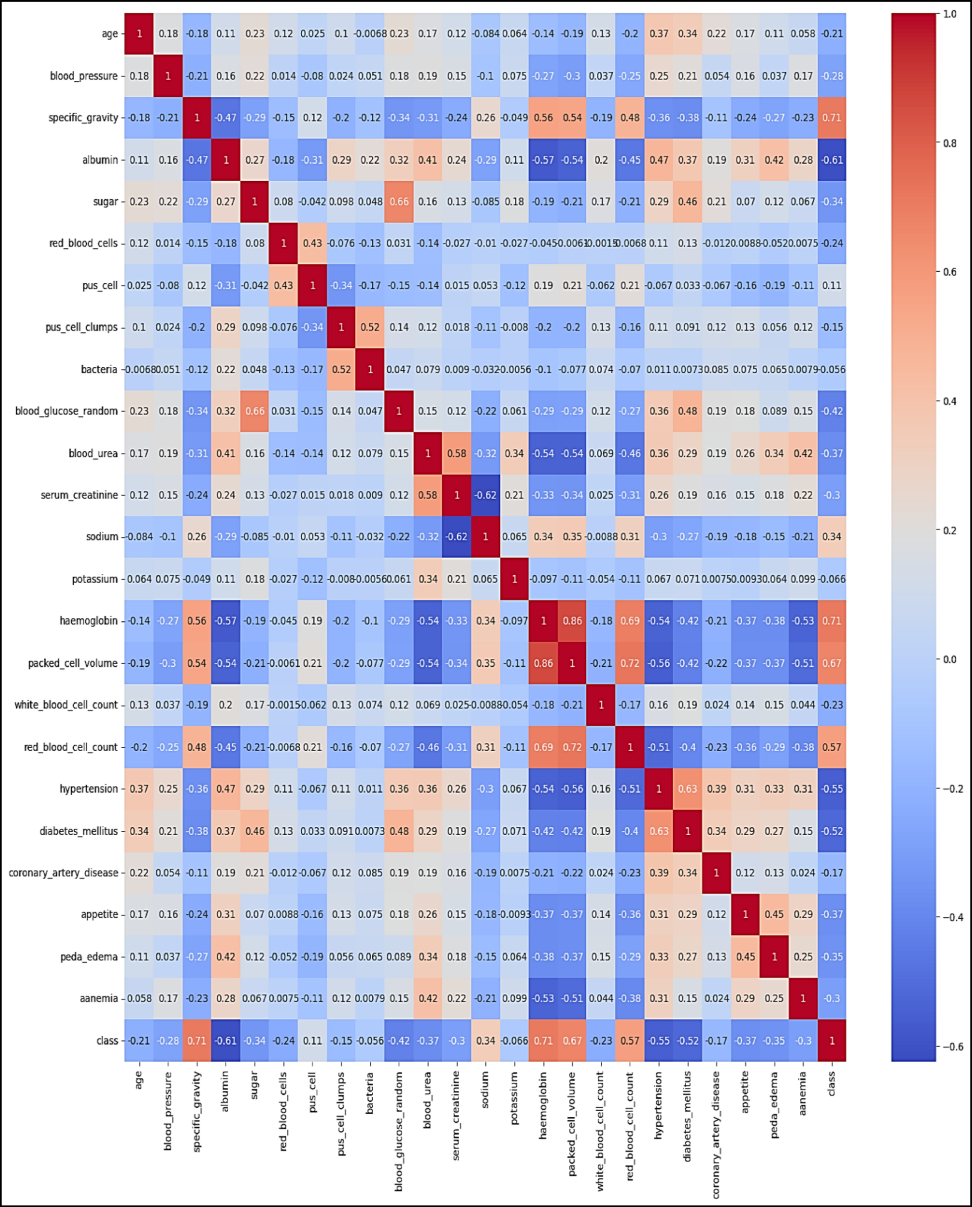
Heatmap analysis for the dataset features.

**Fig. 4:**
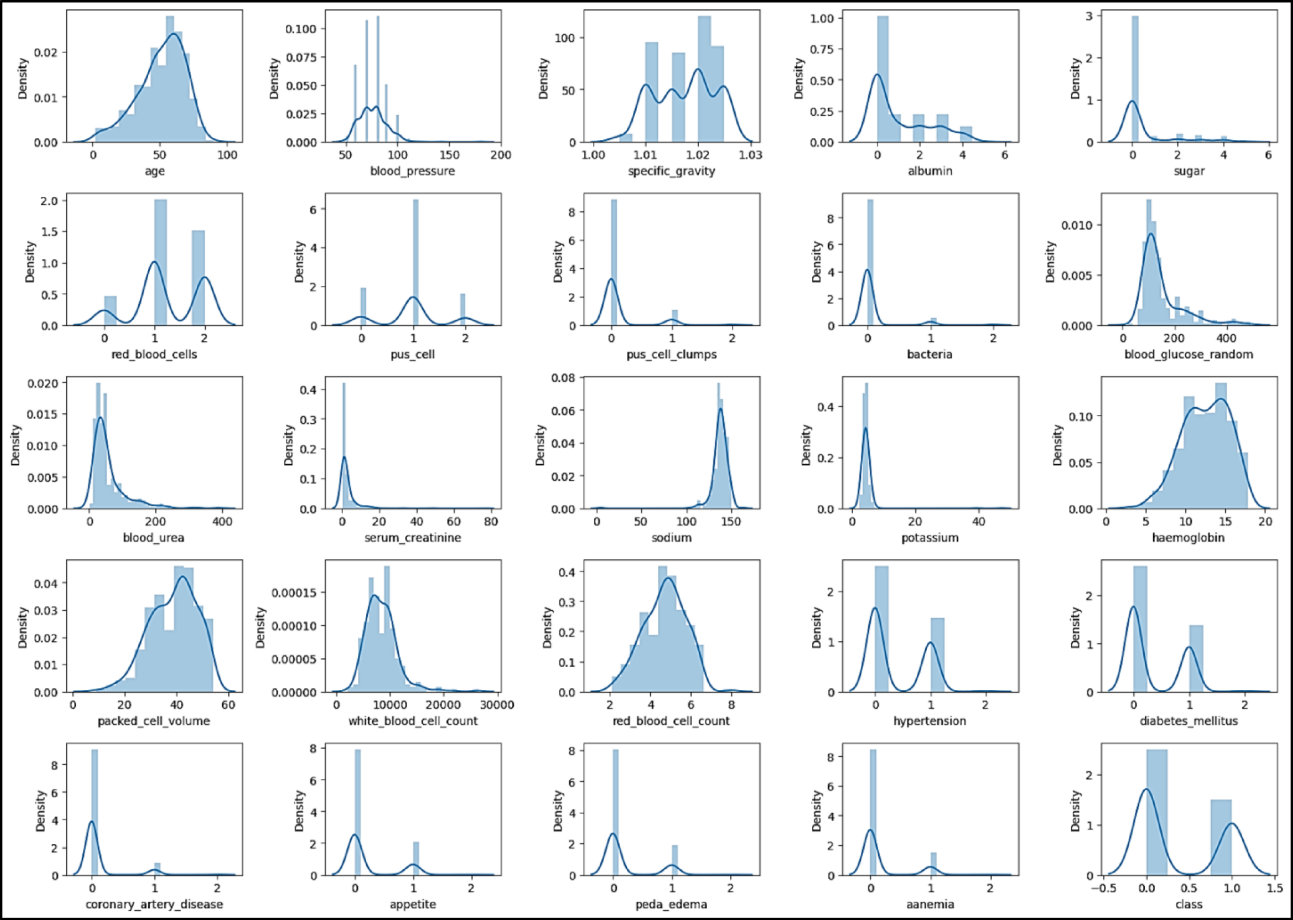
Histogram distribution analysis for the dataset features.

Figure 3 displays a heatmap analysis of the dataset features. Heatmap analysis is a useful approach for visually illustrating correlations among different features in a dataset. This method is commonly used in exploratory data analysis to help identify patterns and relationships between variables. By observing the heatmap, analysts can understand the strength and direction of correlations between features. This helps in grasping how variables interact and may reveal dependencies within the dataset. In summary, heatmap analysis provides a simple yet effective way to uncover underlying structures and associations in complex datasets. Figure 4 showcases the histogram distribution analysis of the dataset features. Examining histogram distributions allows for the visualization of how data points are spread out across different intervals. By studying the shape of the histogram, one can identify trends such as skewness, multimodality, or symmetry within the dataset.

**Table 1:** Statistical analysis for the dataset features.

	Count	Mean	Std	Min	50%	Max
Age	400.0	51.265500	17.073910	2.000	54.000	90.00
Blood_pressure	400.0	76.295000	13.566248	50.00	80.000	180.0
Specific_gravity	400.0	1.017142	0.005470	1.005	1.016	1.025
Albumin	400.0	1.047000	1.281756	0.000	0.600	5.000
Sugar	400.0	0.465000	1.045997	0.000	0.000	5.000
Red_blood_cells	400.0	1.262500	0.655491	0.000	1.000	2.000
Pus_cell	400.0	0.972500	0.593823	0.000	1.000	2.000
Pus_cell_clumps	400.0	0.125000	0.360138	0.000	0.000	2.000
Bacteria	400.0	0.075000	0.299331	0.000	0.000	2.000
Blood_glucose_random	400.0	149.87200	76.73209	22.00	122.0	490.0
Blood_urea	400.0	57.678000	49.562082	1.500	44.00	391.0
Serum_creatinine	400.0	3.077375	5.631741	0.400	1.400	76.00
Sodium	400.0	137.51425	9.282191	4.500	138.0	163.0
Potassium	400.0	4.574750	2.826800	2.500	4.400	47.00
Hemoglobin	400.0	12.576650	2.761359	3.100	12.85	17.80
Packed_cell_volume	400.0	38.965000	8.364238	9.000	40.10	54.00
White_blood_cell_count	400.0	8482.4500	2591.071	2200	8400	26,400
Red_blood_cell_count	400.0	4.707250	0.889911	2.100	4.800	8.000
Hypertension	400.0	0.377500	0.495588	0.000	0.000	2.000
Diabetes_mellitus	400.0	0.352500	0.488713	0.000	0.000	2.000
Coronary_artery_disease	400.0	0.095000	0.310186	0.000	0.000	2.000
Appetite	400.0	0.210000	0.413918	0.000	0.000	2.000
Peda_edema	400.0	0.195000	0.402965	0.000	0.000	2.000
Anemia	400.0	0.155000	0.369210	0.000	0.000	2.000
Class	400.0	0.375000	0.484729	0.000	0.000	1.000

### Z-score normalization

Normalization is a widely used preprocessing technique in machine learning. It’s employed particularly when the dataset exhibits significant variability. The goal of normalization is to analyze all elements within a consistent framework, allowing for comparisons across different scales. Mathematical functions are utilized to transform data from various scales into a standardized scale. This process involves managing the minimum and maximum values of the data, thereby standardizing the range. The aim is to assign a value of 0 to the smallest data point and a value of 1 to the largest, while evenly distributing all other values between this range of 0 to 1. The application of the minimum-maximum formula, also known as Z-score normalization, is demonstrated in Eq. (1).

1 $$\:z=\frac{x-{\upmu\:}}{\sigma\:}$$

where $\:z$ is the transformed data, and $\:x$ represents the input value. $\:{\upmu\:}$ is the mean of the data and $\:\sigma\:$ is the standard deviation of the data.

### Machine learning classifiers models

Our study delved into a range of machine learning classifiers using our training dataset. We explored both linear and non-linear algorithms. Recent studies have highlighted the effectiveness of tree-based bagging and boosting classifiers over traditional models like neural networks. Our analysis included various sub-classifiers such as extra trees (ET), random forest (RF), decision tree (DT), bagging classifier (BC), adaptive boosting (AdaBoost), and k-nearest neighbor (KNN).

#### Extra trees (ET) model

Extra trees (ET), also known as extremely randomized trees, is a tree-based ensemble that is comparable to random forest^42^. This model, which is somewhat new within the discipline of machine learning, is similar to an extension of the popular random forest technique. Its purpose is to reduce the likelihood of over fitting^43^. Using the traditional top-down method, the extra trees model constructs an assembly of un-pruned decisions or regression tree structures. Its two primary distinctions from previous tree-based ensemble approaches are that it grows the trees using the entire learning sample (instead of a bootstrap replica) and separates nodes by selecting cut-points totally at random. The extra trees procedure trains each base estimator using a selected set of features, much like the random forest. It refrains from choosing a feature in addition to its matching value at random for node splitting. Several decision trees are combined to produce a more robust model using ensemble learning model called extra trees (ET)^44^. ET divides the node using a randomly selected set of features for every tree and a randomized threshold value for every feature.

#### Random forest (RF) model

Random forest (RF) model, which can be utilized in batching or non-stream training for problems like regression and classification^45^. Several decision trees are grown by the algorithm and then combined into one single model. The ultimate prediction produced by the model for the classification issues is determined by the majority of votes cast by each tree, each of which predicts a class. To prevent overfitting and provide a robust prediction, the basic principle underlying RF is to create a forest of trees with minimal or no connections with one another. There are many distinct trees in the forest that can make accurate predictions, even if some of them are noise-sensitive and inaccurate^46^. The overall percentage of the trees’ votes is less likely to overfit, and the anticipated error is less if the decisions made by the trees have not been correlated. Random feature selection and the process of bagging (bootstrap aggregating) are the two techniques that the model employs while constructing each individual tree in order to reduce the associations between the trees and boost the diversity in the forest^47^. To get over a single classifier’s constraints in generating the best answer, RF is a resilient ensemble learning (EL) approach made up of many decision tree classifiers. Thus, an RF method that employs the majority vote approach and incorporates several trees as opposed to a single tree is used to determine the final class label.

#### Decision tree (DT) model

Decision tree (DT) is a straightforward to use, non-parametric structure classification technique that can regulate nonlinear relationships between classes and attributes^48^. A method for building a model of prediction that makes use of the tree structure to address classification issues is the Inter-DT method. Specifically, DT represents a tree-based structure of instructions and may be characterized as a process that recursively divides the data that is input into progressively smaller subsets^49^. An array of thresholds defined for each of the inner nodes of the tree serves as the basis for the splitting procedure. Internal nodes divide input data into sub-nodes from the root node, which is the initial node in the DT, and then further divide sub-nodes into more sub-nodes. The data entered are classified using this incrementally binary subdivision, where the final nodes, referred to as leaf nodes (leaves), represent the final target classes^50^. The use of DTs has some issues, where the two most significant ones are overfitting and producing a suboptimal solution^51^. Because a DT just uses one tree, it could not yield the best final model. When utilizing DT, overfitting is another prevalent issue that must be considered. The inner nodes (decision nodes) of the data arrangement within the tree are used to test the qualities of the data, while the edges branch the test results, and the leaves indicate the problem’s class labels. By choosing the most useful data characteristic for dividing the data into every node, the DT construction algorithm finds ways to partition the training set^52^. The construction technique can be employed within the train phase in which it begins execution at the tree’s root, which holds the entire dataset.

#### Bagging classifier (BC) model

Bagging classifier is a model for building classifier ensembles that are conceptually similar but fundamentally different^53^. Bagging was developed by Breiman^54^, which was then expanded to Arcing to support the adaptive incremental generation of the ensemble that forms the basis of the Boosting approach. Using bootstrap sampling to generate random samples from the data set, bagging builds an ensemble of classifiers by creating one classifier for each bootstrap sample. The final decision of classification for an unlabeled data x has been determined through selecting a majority vote among the class labels generated using the L models^54^. The true strength of Bagging is for unstable classifiers like decision trees and neural networks. Small changes in the data set might cause unstable classifiers to become sensitive^55^. As a consequence, two marginally distinct sets of training data used to train the same classifying model might produce very different classifiers. Although the classifiers’ overall accuracies may be comparable, there will be a natural ensemble variety because of the differences in the parameters (such as the neural network’s weights).

#### Adaptive boosting (AdaBoost) model

Adaptive boosting, often known as AdaBoost, is the most widely used boosting algorithm^56^. AdaBoost also needs less algorithmic parameter adjusting than other boost algorithm versions. An AdaBoost model for classification starts by fitting a copy of the original dataset using a reproduce of the previous classifier that has been updated to remove error-prone and inaccurate data points. This allows the subsequent classifiers to concentrate on the cases that lead to greater inaccuracy^57^. AdaBoost is able to produce a hypothesis based on potential labels. The error in prediction of the weak assumption ought to be smaller than 0.5 during training^58^. Choosing the “hard” trained data and samples given to the following iteration is the aim of distribution. By using weighted majority voting to determine the classes from each individual hypothesis’ prediction, AdaBoost creates an ensemble and a collection of hypotheses. AdaBoost is a kind of iterative computation whose basic idea is to train several classifiers (that is, weak learners) using a preparation set, and then use several different coordination techniques to create an additional robust model.

#### K-Nearest neighbor (KNN) model

K-nearest neighbors (KNN) stand out as a straightforward, yet robust algorithm employed in both classification and regression tasks^59^. In classification, it assigns a label to a data point by assessing the majority class among its K closest neighbors within the feature space. Unlike many other algorithms, KNN doesn’t undergo an explicit training phase^60^. Instead, it just remembers the training data. When presented with a new data point for classification, KNN identifies its K nearest neighbors based on a chosen distance metric, typically the Euclidean distance. Once the K nearest neighbors is identified, KNN assigns the class label to the new data point by conducting a majority vote among its neighbors. The class that occurs most frequently among the K neighbors becomes the predicted class for the new data point. The selection of the parameter K, representing the number of neighbors to consider, holds significance in KNN. A smaller K value enhances sensitivity to noise and outliers, while a larger K value might lead to smoothing out decision boundaries, potentially resulting in underfitting^61^. Determining the optimal K value often entails employing cross-validation or other tuning techniques. The choice of distance metric greatly influences KNN’s performance. While the Euclidean distance is widely used, alternative metrics such as Manhattan distance, Minkowski distance, or cosine similarity may better suit the data characteristics.

### Explainable artificial intelligence

Explainable artificial intelligence (XAI) refers to the set of techniques and methods aimed at making artificial intelligence models and their decisions understandable and interpretable to humans. As AI systems become increasingly prevalent in critical applications such as healthcare, finance, and criminal justice, the need for transparency and accountability in AI decision-making has grown significantly. The main goal of this research is to develop classification pipelines and improve the interpretability of the resulting predictions. The intensity of red in the plot corresponds to the magnitude of the feature value^62^. In this study, we utilized a tool called shapley additive explanations (SHAP) to elucidate various classifiers. SHAP is a widely used method in explainable AI for interpreting game theoretical classifiers^15^. It evaluates shapley values for each feature to understand their impact on predictions. The SHAP plot provides a detailed overview of the classifiers’ insights on a global scale. Each dot on the plot represents an individual data point, while the features are listed along the y-axis in descending order. SHAP values are depicted on the x-axis, and the color gradient indicates the feature values.

### K-Nearest neighbor (KNN) imputer

The k-nearest neighbor (KNN) imputer is a method utilized to fill in missing values within a dataset by considering the values of its closest neighboring data points^63^. The underlying principle is that data points resembling each other tend to possess similar feature values. When encountering a missing value in the dataset, the KNN imputer locates the K nearest neighbors to the data point containing the missing value. These neighbors are determined based on a distance metric like Euclidean distance. After identifying the nearest neighbors, the KNN imputer computes the average (or weighted average) of the feature values associated with the missing value across these neighboring data points. Subsequently, this calculated value fills in the missing value within the dataset^64^. The parameter K, representing the number of neighbors considered, plays a critical role in KNN imputation. Opting for a smaller K value may result in imputed values closely resembling those of the nearest neighbors, while a larger K value may yield a smoother imputation, albeit potentially introducing noise from more distant neighbors. KNN imputation extends its applicability to categorical variables by treating them as discrete points within a multidimensional space^65^. In this scenario, instead of computing averages, the mode (most frequent value) of the categorical feature across the nearest neighbors is employed for imputation.

### Binary Breadth-First search (BBFS)

Binary breadth-first search (BBFS) is a feature selection algorithm that is often used in the context of binary classification problems^5^. Its objective is to identify and select the most relevant features from a given set of features to improve the performance of a machine learning model^66^. The algorithm is based on the principles of breadth-first search (BFS), a graph traversal algorithm, and it efficiently explores the feature space^67^. The mathematical algorithm for binary breadth-first search for feature selection is illustrated in Algorithm 1.

**Algorithm 1:**
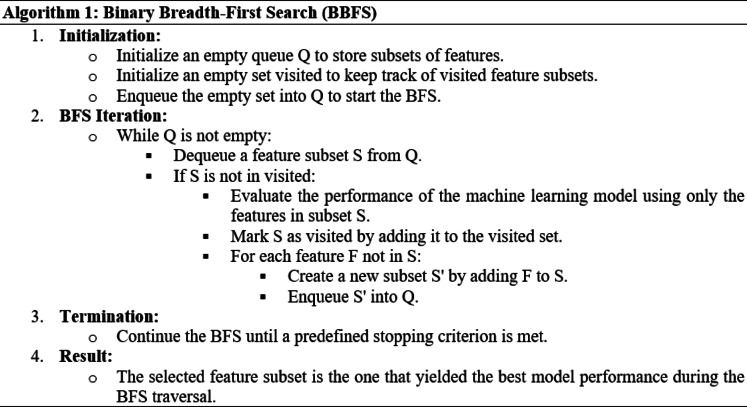
Binary Breadth-First Search (BBFS).

Algorithm 1 systematically explores the feature space in a breadth-first manner, ensuring that the selected subset is chosen based on the performance of the model used. The stopping criterion helps control the search space and prevent exhaustive exploration.

### Evaluation metrices

In this study, five evaluation metrics: accuracy, sensitivity, specificity, F-score, and area under the ROC curve (AUC) are utilized to assess the performance of the models. These evaluation criteria, outlined from Eq. 1 to Eq. 5, serve as benchmarks for evaluating the models. During both the training and testing phases of data classification problems, these evaluation metrics are applied^68^. In the training phase, the assessment metric serves as a discriminator, aiding in the selection of the most effective classifier for future assessments. Conversely, during the testing phase, the evaluation metrics act as evaluators, gauging the classifier’s performance when confronted with unknown data.

2 $$\:{\text{Accuracy}}\:{\text{ = }}\frac{{{\text{TP + TN}}}}{{{\text{TP + TN + FP + FN}}}}$$

3 $$\:\text{S}\text{e}\text{n}\text{s}\text{i}\text{t}\text{i}\text{v}\text{i}\text{t}\text{y}\:=\frac{TP}{TP+FN}$$

4 $$\:\text{S}\text{e}\text{n}\text{s}\text{i}\text{t}\text{i}\text{v}\text{i}\text{t}\text{y}\:=\frac{TP}{TP+FN}$$

5 $$\:\text{F}-\text{s}\text{c}\text{o}\text{r}\text{e}=2*\frac{Precision*Recall}{Precision+Recall}$$

6 $$\:AUC=\frac{{S}_{p}-{N}_{p}+({N}_{n}+1)/2}{{N}_{p}{N}_{n}}$$

where $\:TP$, $\:TN$, $\:FP$, $\:FN$ represent true positive, true negative, false positive, and false negative values, respectively. $\:{S}_{p}$ is the proportion of correctly classified cases belonging to the negative class, and $\:{N}_{p}$ $\:{\text{a}\text{n}\text{d}\:N}_{n}$ are the number of positive and negative classes, respectively.

## Results and discussion

The experiments were conducted using Jupyter Notebook version 6.4.6, a widely used tool for Python-based data analysis and visualization. Jupyter Notebook provides a robust platform for coding, executing, and documenting processes, along with generating graphical representations of data. This versatile tool supports multiple programming languages, including Python 3.8, and is accessible via a web browser. The experiments were performed on a Microsoft Windows 10 computer equipped with an Intel Core i5 CPU and 16 GB of RAM. In this study, the explainable artificial intelligence-CKD (XAI-CKD) model is introduced for the process of classification. XAI-CKD applies explainable AI (XAI) by utilizing extra trees (ET) and SHAP (shapley additive explanations) values. Also, binary breadth-first search (BBFS) algorithm is used to select the most important features for XAI-CKD model. The selected features using BBFS algorithm are specific gravity, red blood cells, albumin, hypertension, hemoglobin, diabetes mellitus, packed cell volume, appetite, peda edema, pus cell, red blood cell count, blood glucose random, sugar, coronary artery disease, age, pus cell clumps, and bacteria. This methodology is designed to derive valuable insights for enhancing decision-making strategies within the field of classifying chronic kidney diseases. The performance of XAI-CKD is compared with another machine learning models, namely, random forest (RF), decision tree (DT), bagging classifier (BC), adaptive boosting (AdaBoost), and k-nearest neighbor (KNN), and the performance of the models is evaluated using accuracy, sensitivity, specificity, F-score, and area under the ROC curve (AUC).

To ensure the robustness of the results, each experiment was conducted over ten independent runs. In each run, the dataset was randomly split into training (70%) and testing (30%) sets, and the models were retrained and evaluated from scratch. Performance metrics such as accuracy, sensitivity, specificity, F-score, and AUC were averaged across these runs to reduce the effects of any variance due to random data partitioning in the training process.

To ensure a fair comparison among all models, we employed grid search with 5-fold cross-validation for hyperparameter tuning. This approach exhaustively searches through specified hyperparameter ranges to identify the best configuration for each model based on validation performance. Table 2 presents the hyperparameter tuning process using grid search with 5-fold cross-validation for each machine learning model. For each model, key hyperparameters were explored over a defined search range based. The table includes the search ranges used during tuning and the optimal values selected based on validation performance. ET model was evaluated with different values for n_estimators ranging from 50 to 200, and criterion hyperparameter was tested with ‘gini’ and ‘entropy’. The optimal configuration selected was n_estimators = 50 and criterion = ‘gini’. Similarly, RF model performed best with n_estimators = 150 and criterion = ‘entropy’. DT model performed best for a randomized splitting strategy and the entropy criterion. BC model achieved optimal results with 50 estimators and max_samples = 1.0. AdaBoost model achieved optimal results with a low learning rate of 0.01 and 100 estimators. KNN model demonstrated optimal results using 30 neighbors and distance weighting.

**Table 2:** Hyperparameter search ranges and optimal configurations for each model.

Model	Hyperparameter	Search Range	Best Value
ET	n_estimators	[50, 100, 150, 200]	50
	criterion	[‘gini’, ‘entropy’]	gini
RF	n_estimators	[100, 150, 200]	150
	criterion	[‘gini’, ‘entropy’]	entropy
DT	splitter	[‘best’, ‘random’]	random
	criterion	[‘gini’, ‘entropy’]	entropy
BC	n_estimators	[10, 50, 100]	50
	max_samples	[0.5, 0.7, 1.0]	1.0
AdaBoost	n_estimators	[50, 100, 150]	100
	learning_rate	[0.01, 0.1, 0.5, 1.0]	0.01
KNN	n_neighbors	[5, 10, 20, 30, 40]	30
	weights	[‘uniform’, ‘distance’]	distance

Table 3 demonstrates the performance of the proposed XAI-CKD model compared to another five traditional machine learning models, namely, random forest (RF), decision tree (DT), bagging classifier (BC), adaptive boosting (AdaBoost), and k-nearest neighbor (KNN) in terms of accuracy, sensitivity, specificity, F-score, and area under the ROC curve (AUC). As depicted in Table 3, the proposed XAI-CKD model achieved the best results with accuracy of 99.9%, sensitivity of 99.9%, specificity of 99.9%, F-score of 99.9% that reflects a balance between precision and recall, and AUC of 1.0 that indicates excellent discrimination ability. The KNN model achieved the worth results with accuracy of 66.6%, sensitivity of 66.7%, specificity of 66.7%, F-score of 66.6% and AUC of 0.673. The second model achieved the best results after the proposed XAI-CKD model is RF model, its accuracy, sensitivity, specificity, F-score, and AUC are 97.5%, 97.5%, 97.5%, 97.4%, and 0.968. The third model achieved best results after the RF model is DT model, its accuracy, sensitivity, specificity, F-score, and AUC are 95.8%, 95.8%, 95.8%, 95.8%, and 0.955. The fourth model achieved best results after the DT model is BC model, its accuracy, sensitivity, specificity, F-score, and AUC are 94.1%, 94.1%, 94.2%, 94.1%, and 0.936. The fifth model achieved the best results after the BC model is Adaboost model, its accuracy, sensitivity, specificity, F-score, and AUC are 85%, 85%, 85.2%, 85.3%, and 0.862.

**Table 3:** Performance of the proposed XAI-CKD model and another traditional machine learning models.

Models	Accuracy	Sensitivity	Specificity	F-score	AUC
XAI-CKD	99.9%	99.9%	99.9%	99.9%	1.000
RF	97.5%	97.5%	97.5%	97.4%	0.968
DT	95.8%	95.8%	95.8%	95.8%	0.955
BC	94.1%	94.1%	94.2%	94.1%	0.936
AdaBoost	85%	85%	85.2%	85.3%	0.862
KNN	66.6%	66.7%	66.7%	66.6%	0.673

Table 4 depicts the performance of the BBFS algorithm against another feature selection algorithms, namely, binary whale optimization algorithm (BWOA), binary particle swarm optimization (BPSO), and binary grey wolf optimizer (BGWO) in terms of accuracy, sensitivity, specificity, F-score and AUC.

**Table 4:** Comparison between the outcomes of BBFS feature selection algorithm and several feature selection algorithms.

Models	Accuracy	Sensitivity	Specificity	F-score	AUC
XAI-CKD-BBFS	99.9%	99.9%	99.9%	99.9%	1.000
XAI-CKD-BWOA	98.01%	98.01%	98.02%	98.01%	0.984
XAI-CKD-BPSO	97.75%	97.75%	97.75%	97.76%	0.979
XAI-CKD-BGWO	95.84%	95.85%	95.84%	95.84%	0.957

From Table 4, the best results are obtained by XAI-CKD-BBFS model with accuracy of 99.9%, sensitivity of 99.9%, specificity of 99.9%, F-score of 99.9% that reflects a balance between precision and recall, and AUC of 1.0 that indicates excellent discrimination ability. The XAI-CKD-BGWO model achieved the worth results with accuracy of 95.84%, sensitivity of 95.85%, specificity of 95.84%, F-score of 95.84% and AUC of 0.957.

Table 5 demonstrates the configuration of the hyperparameters for the feature selection algorithms used in this study.

**Table 5:** Hyperparameters for the feature selection algorithms.

Algorithm	Hyperparameters	Values
BBFS	Iterations Number of vertices (*V*) Number of edges (*E*)	100 10 15
BWOA	Iterations Population size	100 150
BPSO	Iterations Acceleration constants Inertia Wmax, Wmin Particles	100 [8, 8] [0.6, 0.9] 50
BGWO	Iterations Wolves	100 50

Table 6 demonstrates the results of median fitness, mean fitness, best fitness, worst fitness, and standard deviation fitness for four algorithms, namely, BBFS, BWOA, BPSO, and BGWO. BBFS achieved the best results in terms of median fitness, mean fitness, best fitness, worst fitness, and standard deviation fitness.

**Table 6:** Performance of BBFS model compared to another algorithms.

Metrics	BBFS	BWOA	BPSO	BGWO
Median fitness	0.42515	0.45538	0.46962	0.47364
Mean fitness	0.44197	0.46887	0.46764	0.47548
Best fitness	0.34377	0.38917	0.44794	0.43958
Worst fitness	0.65371	0.71512	0.73646	0.74154
Standard deviation fitness	0.22427	0.27967	0.27944	0.28168

**Fig. 5:**
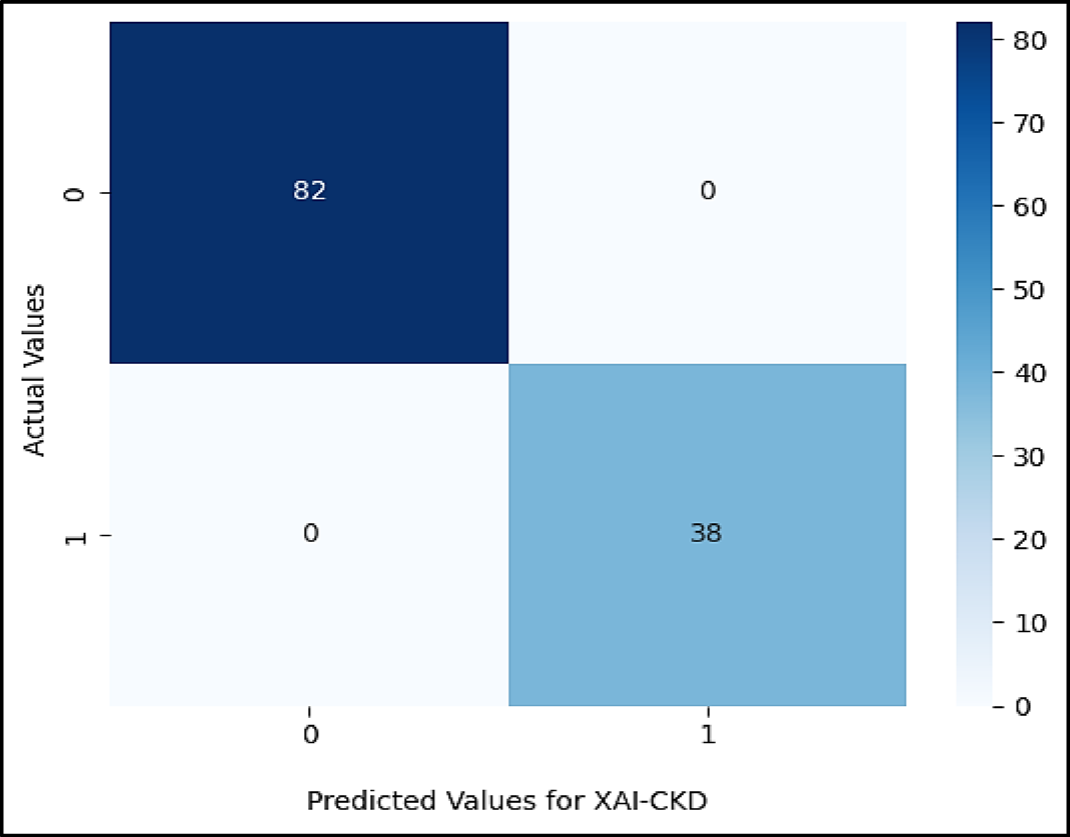
Confusion matrix for the proposed XAI-CKD model.

**Fig. 6:**
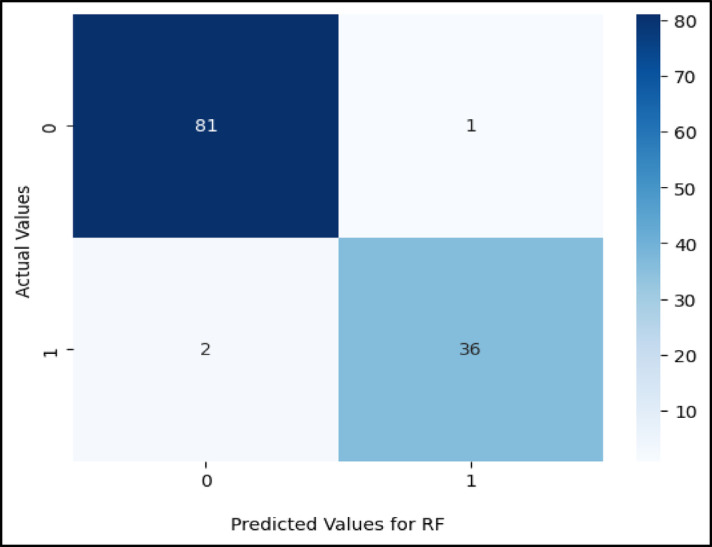
Confusion matrix for RF model.

**Fig. 7:**
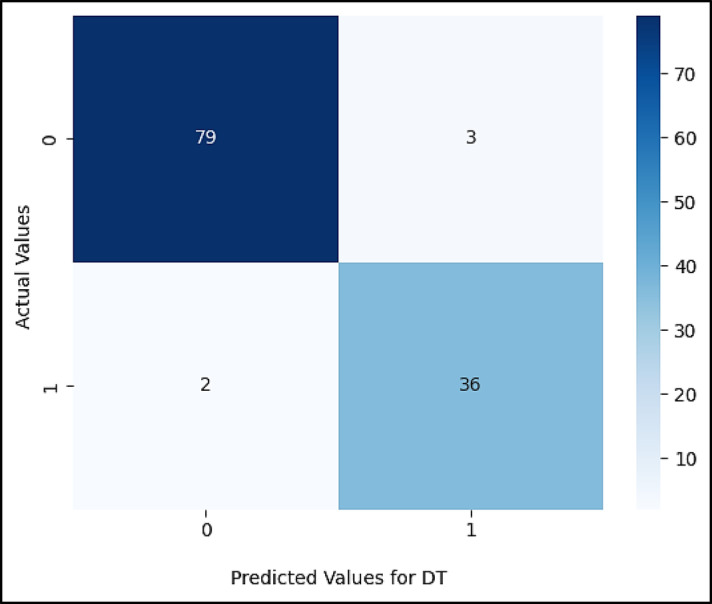
Confusion matrix for DT model.

**Fig. 8:**
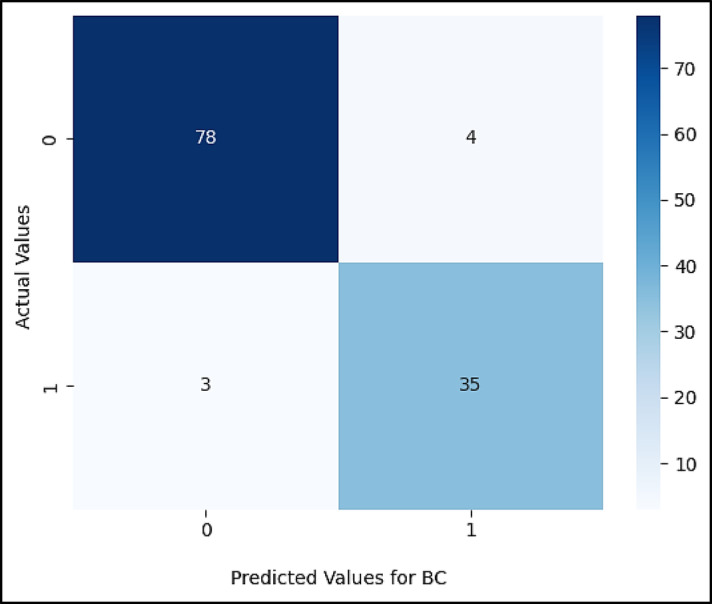
Confusion matrix for BC model.

**Fig. 9:**
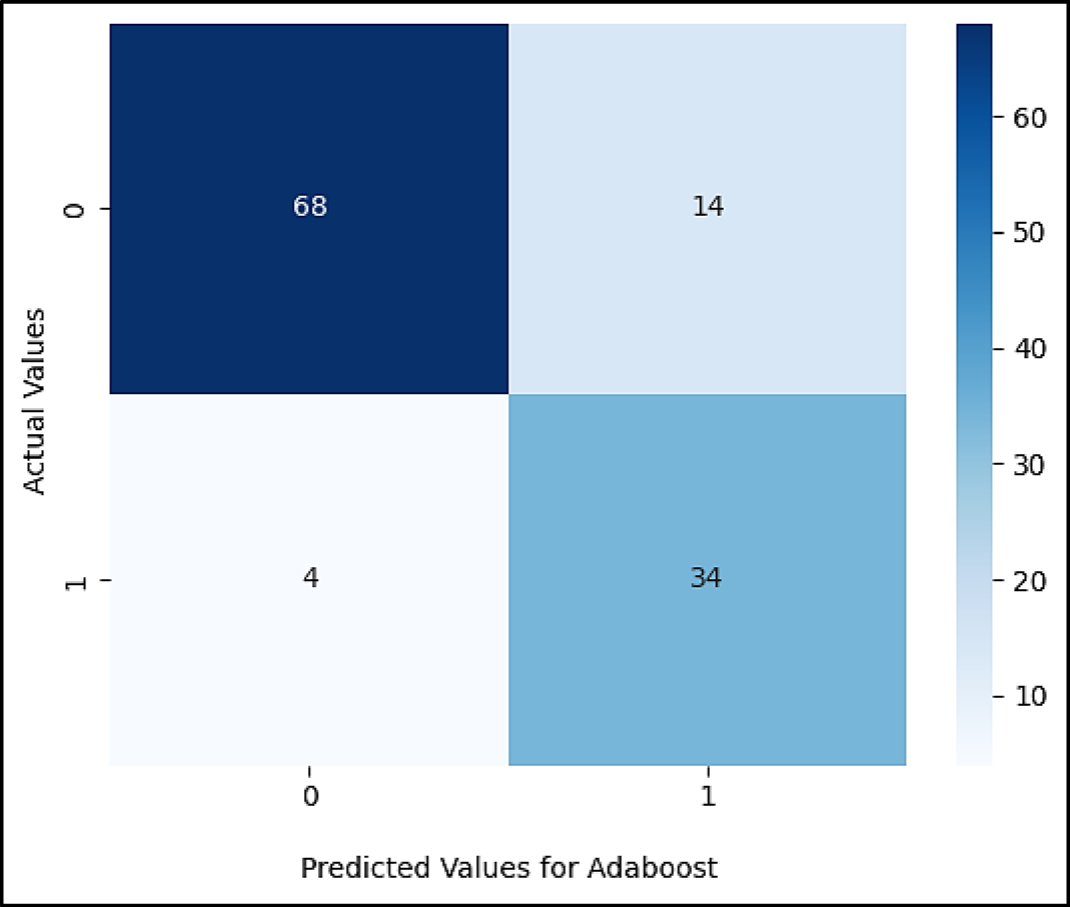
Confusion matrix for Adaboost model.

**Fig. 10:**
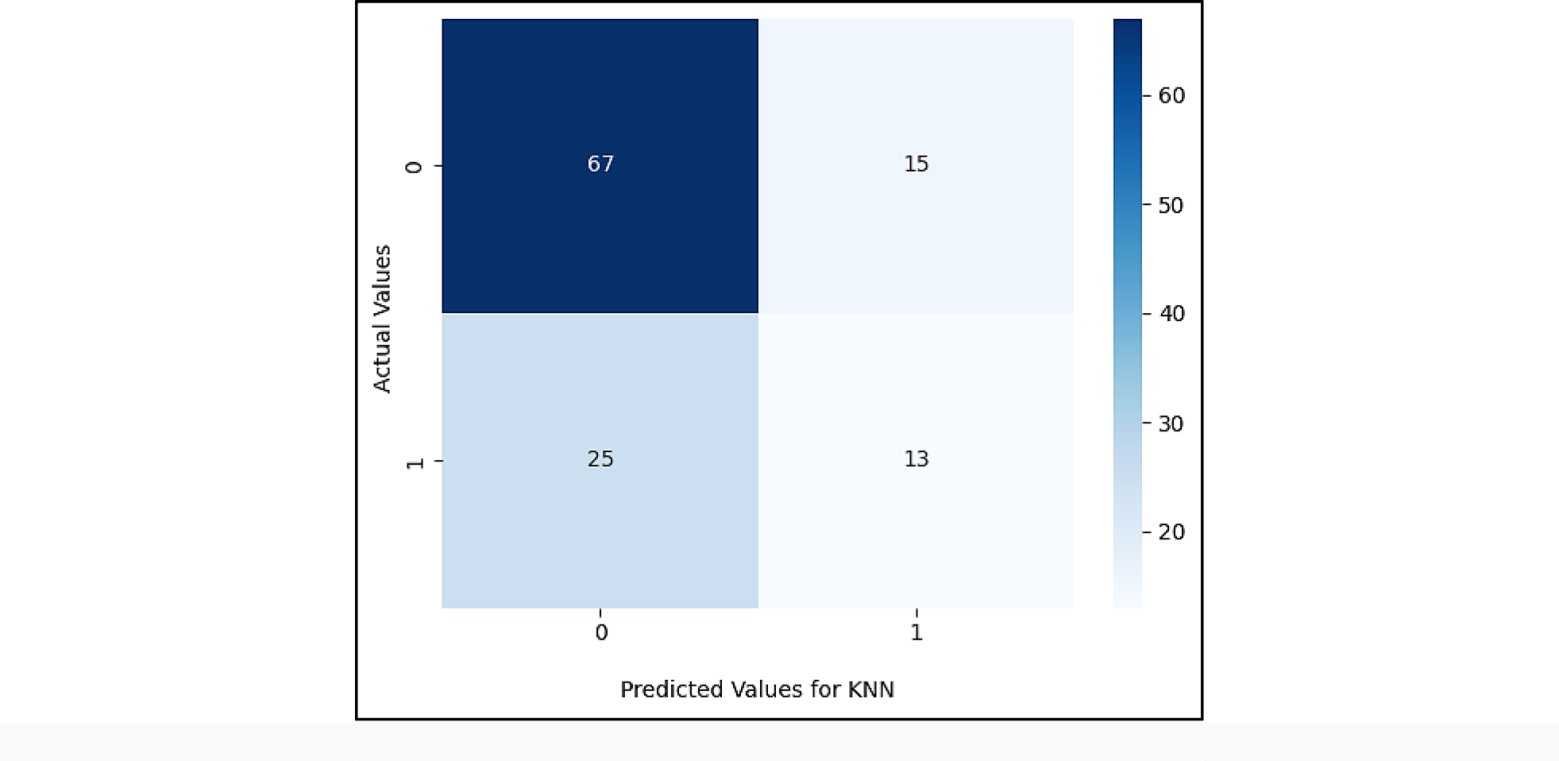
Confusion matrix for KNN model.

Figures (5, 6, 7, 8, 9,10) demonstrate the confusion matrix for the proposed XAI-CKD, random forest (RF), decision tree (DT), bagging classifier (BC), adaptive boosting (AdaBoost), and k-nearest neighbor (KNN) models during the testing phase.

**Fig. 11:**
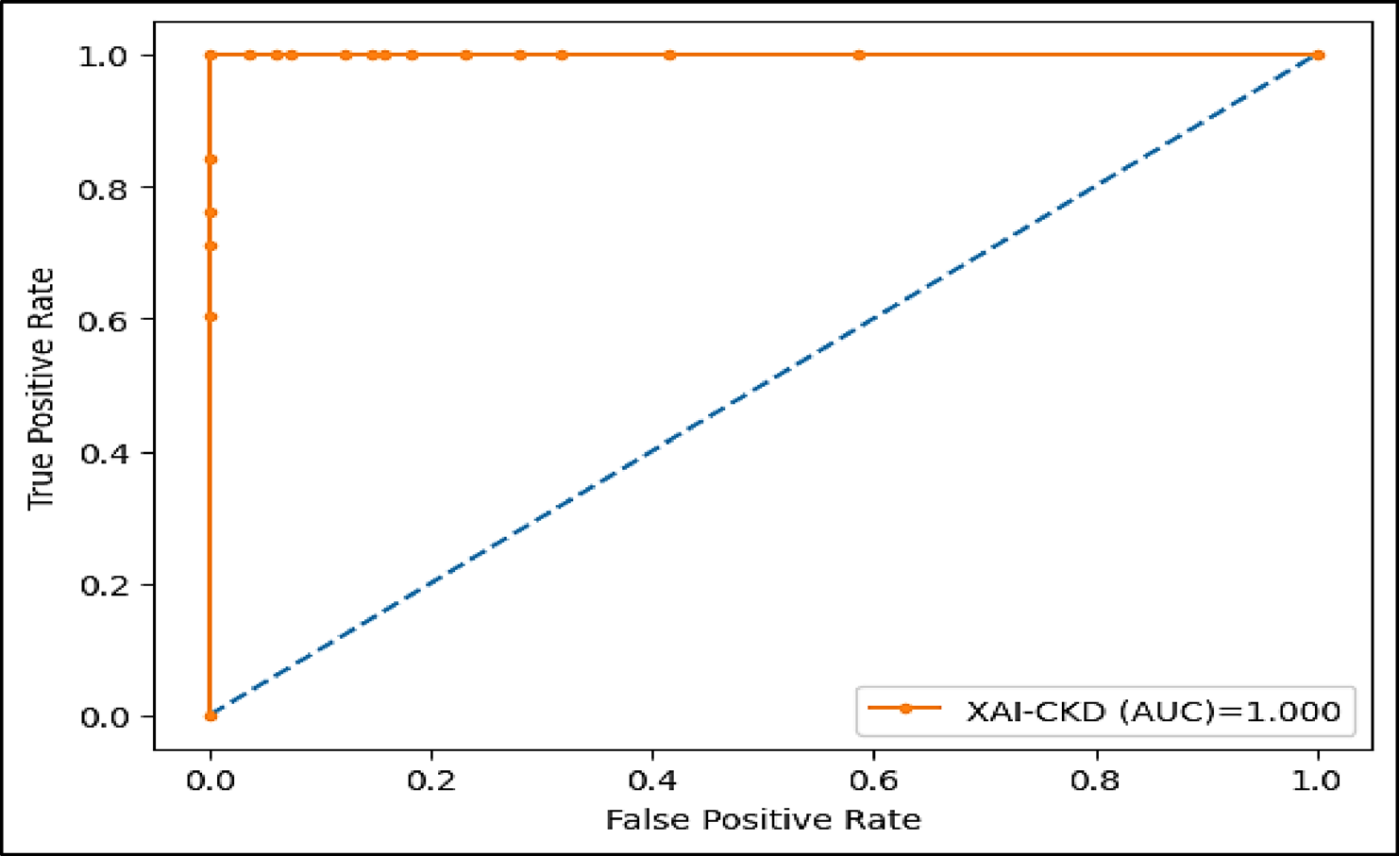
AUC for the proposed XAI-CKD model.

**Fig. 12:**
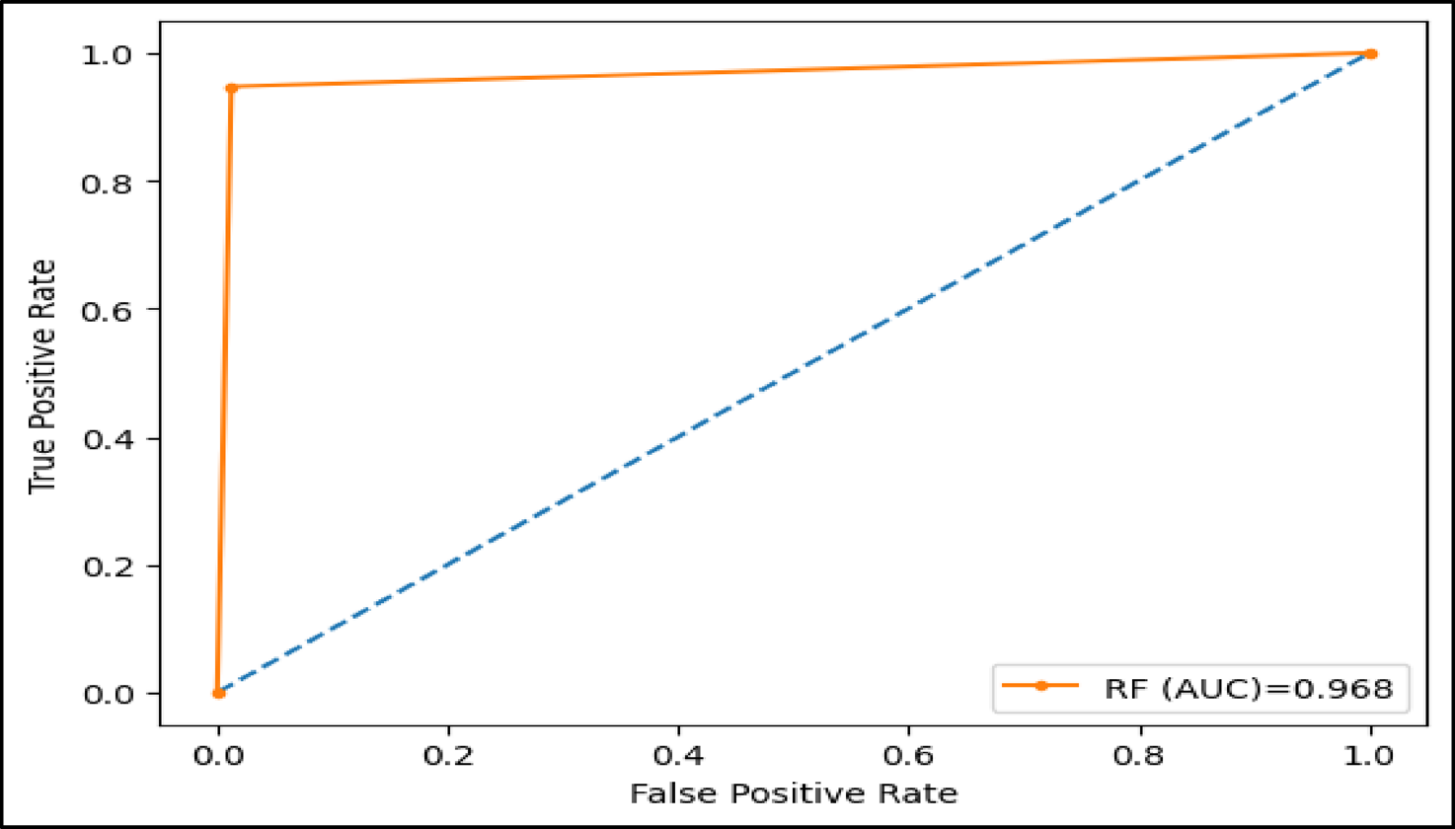
AUC for RF model.

**Fig. 13:**
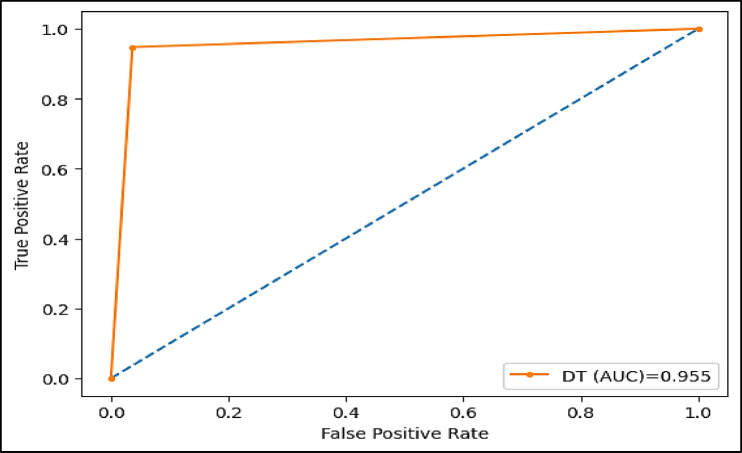
AUC for DT model.

**Fig. 14:**
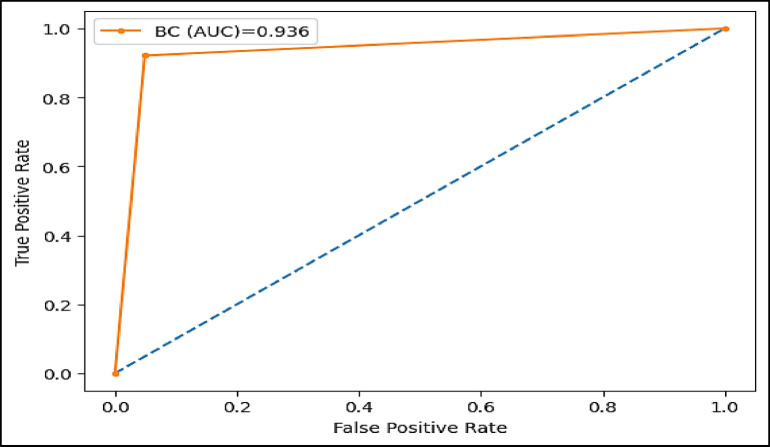
AUC for BC model.

**Fig. 15:**
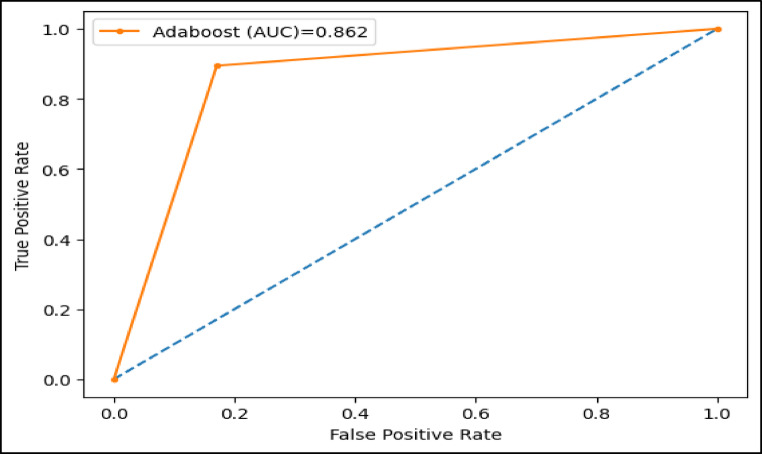
AUC for Adaboost model.

**Fig. 16:**
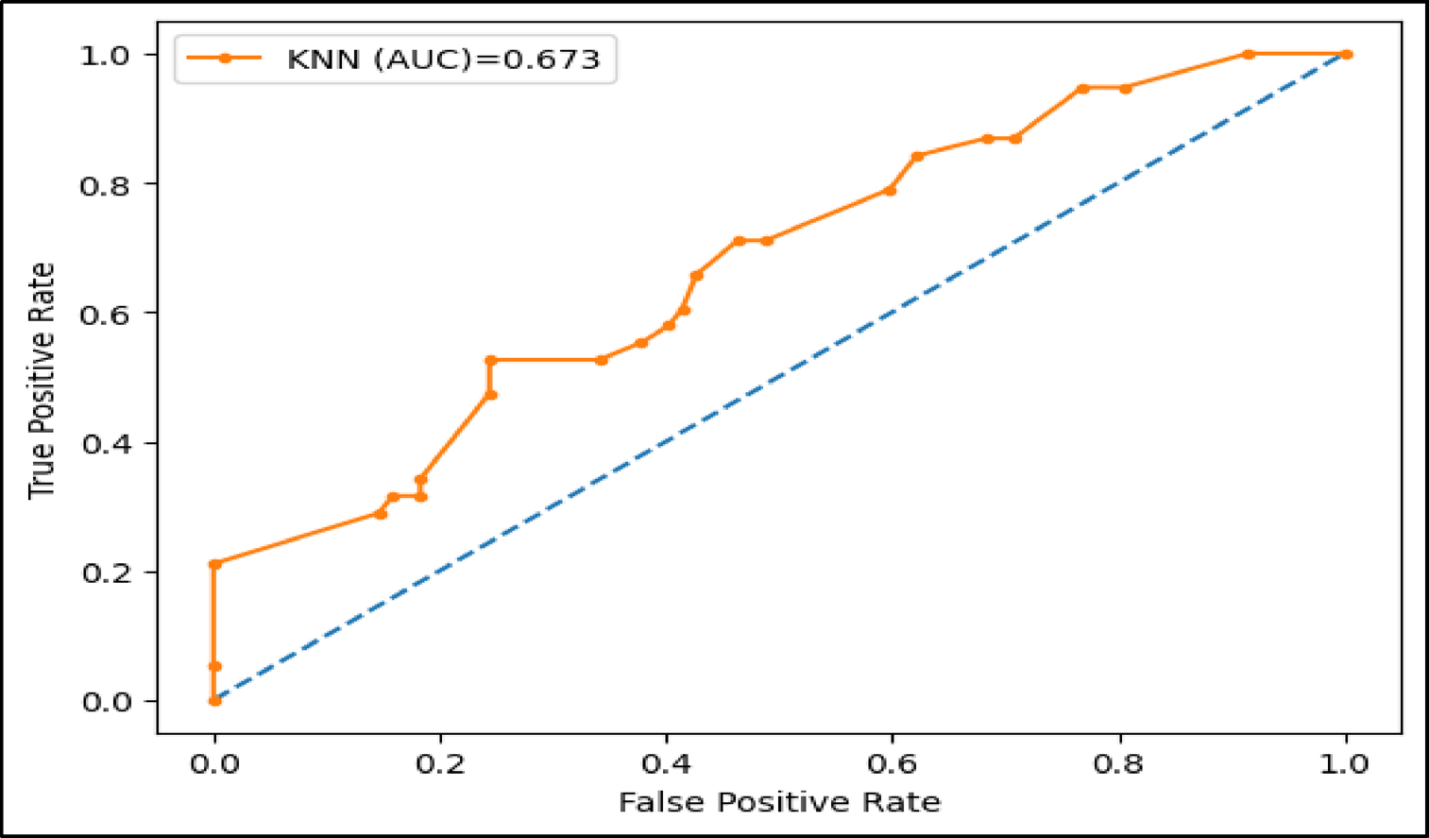
AUC for KNN model.

The area under the ROC curve (AUC), is determined by plotting the receiver operating characteristic (ROC) curve, illustrating how a model’s true positive rate (sensitivity) compares to its false positive rate (1-specificity) at different decision thresholds. A higher AUC indicates a more effective model. Figures (11–16) showcase the AUC values for the models XAI-CKD, random forest (RF), decision tree (DT), bagging classifier (BC), adaptive boosting (AdaBoost), and k-nearest neighbor (KNN) models during the testing phase. Notably, the XAI-Med model exhibits an AUC of 1.0, which is considered excellent.

Table 7 demonstrates the selected features using BBFS algorithm.

**Table 7:** Selected features using BBFS algorithm.

Features names
age
specific_gravity
albumin
sugar
red_blood_cells
pus_cell
pus_cell_clumps
bacteria
blood_glucose_random
haemoglobin
packed_cell_volume
red_blood_cell_count
hypertension
diabetes_mellitus
coronary_artery_disease
appetite
peda_edema

**Fig. 17:**
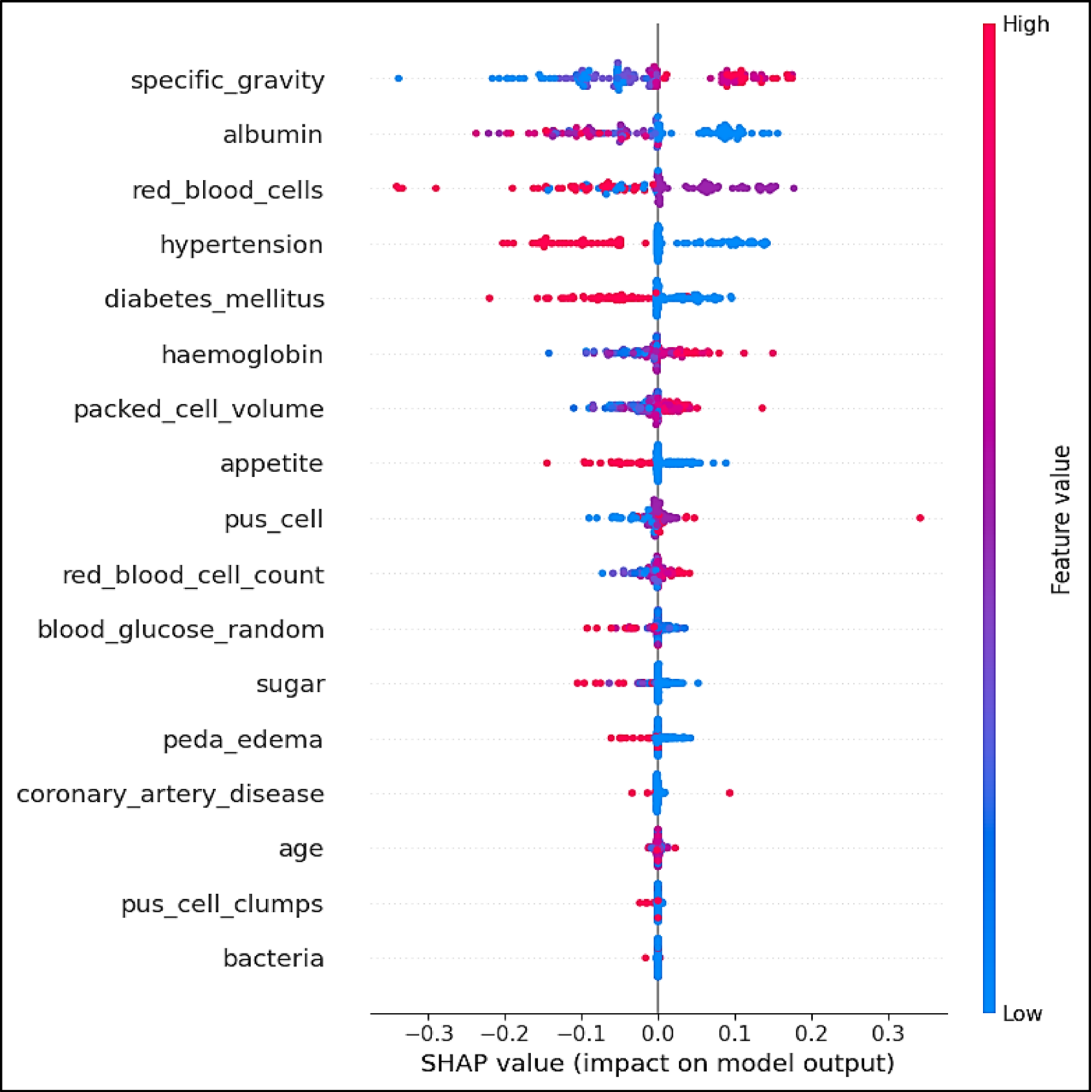
Contribution of the features using XAI- CKD model for chronic kidney disease classification.

Figure 17 illustrates the significance of different features in CKD classification, utilizing the XAI-CKD model. We employed the extra tree (ET) model to compute SHAP (shapley additive explanations) values, aiming to provide deeper insights for improved decision-making in the classification process, as depicted in the Fig. 17. These SHAP summary plots, generated through the ET approach, offer valuable insights crucial for decision-making processes in CKD classification. They visually represent the contribution of each input feature towards the classification, illustrating both the average contribution and the potential range of contributions for each feature. This visualization method provides a clear understanding of the relationships between input variables and the resulting classification, helping identify the most influential factors. Features are ranked on the y-axis based on their average absolute SHAP values, indicating their importance in the model’s predictions. On the x-axis, SHAP values themselves are represented. Positive SHAP values for a feature indicate its presence drives the model’s prediction towards a positive outcome (diagnosis of CKD), while negative SHAP values suggest a tendency towards a negative outcome. Red dots represent instances where an individual is diagnosed with CKD (a positive outcome), while blue dots indicate a tendency towards a negative diagnosis.

The analysis of the SHAP plots provides a clearer explanation of how specific features influence CKD predictions. Higher blood pressure strongly increases the likelihood of CKD, as shown by the SHAP values. This finding aligns with hypertension is well-known role in damaging kidney function, making it a primary risk factor for CKD. Elevated serum creatinine levels emerged as a significant predictor of CKD. This result reflects its clinical importance as an indicator of the reduced glomerular filtration rate (GFR), and a key criterion for CKD diagnosis. High albumin levels consistently pushed predictions toward a CKD classification. This highlights its role as an early biomarker of kidney damage and emphasizes its diagnostic value. Elevated blood pressure and serum creatinine underline the importance of early detection and management to slow CKD progression. Anemia indicators like hemoglobin and packed cell volume reinforce the need for tailored clinical strategies to manage anemia, a common complication in advanced CKD. Blood glucose and hypertension demonstrate the model’s ability to identify systemic conditions, such as diabetes and hypertension, which are leading causes of CKD. This insight can support a lot of comprehensive risk assessments.

**Fig. 18:**
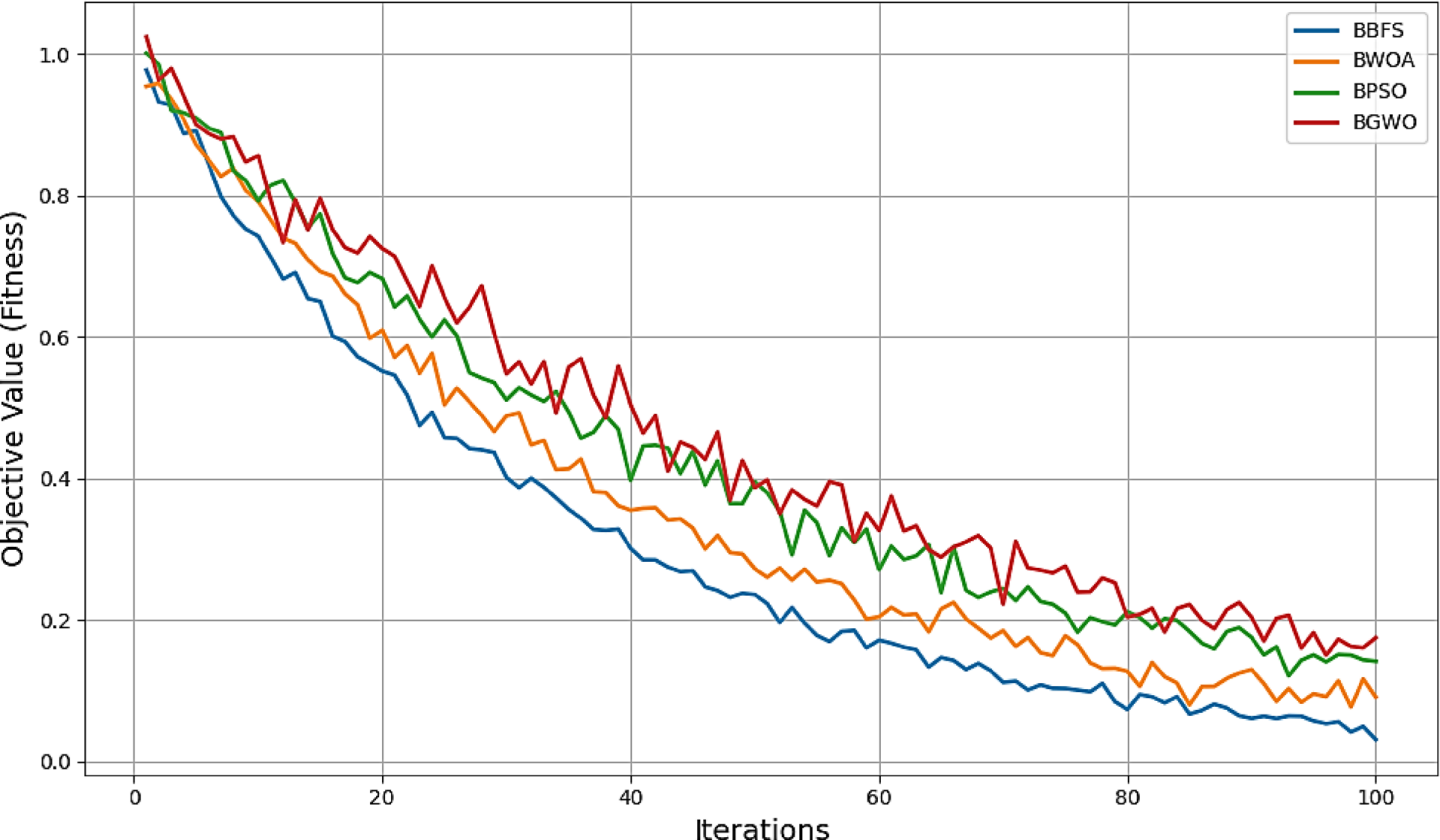
Convergence plots of the optimization algorithms.

Figure 18 demonstrates the convergence curves of feature selection algorithms used in this study.

**Fig. 19:**
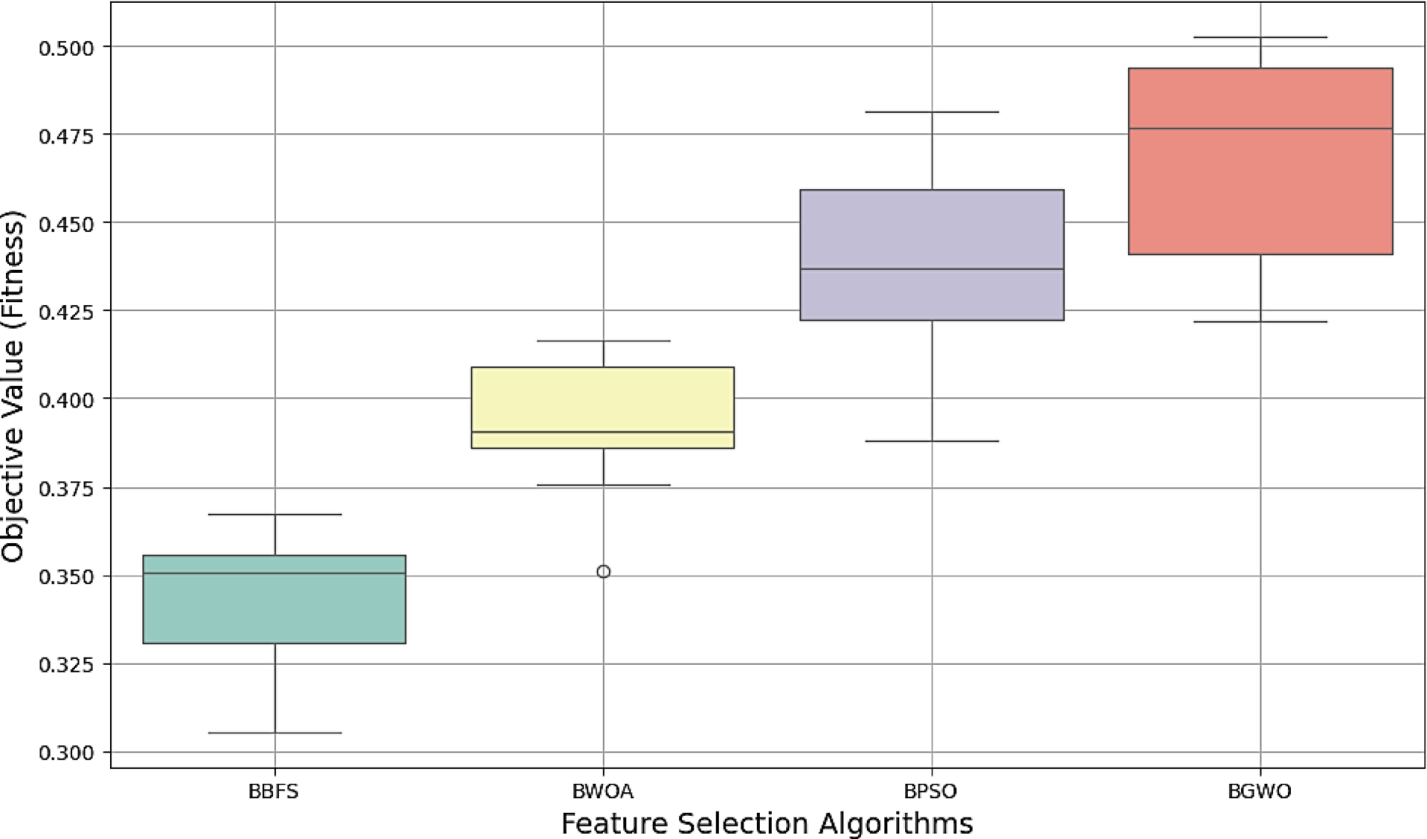
Box plots of objective values for the optimization algorithms.

Figure 19 presents the box plot of the objective values across 10 runs for feature selection algorithms used in this study.

### SHAP analysis and clinical relevance

The SHAP values to identify the features with the greatest impact on CKD classification. Our findings highlight the following features as the most important: specific gravity, albumin, hemoglobin, packed cell volume, red blood cell count, blood glucose random, and hypertension. These features are highly relevant in predicting CKD and align closely with established clinical knowledge. Specific gravity and albumin are essential markers of kidney function. Specific gravity indicates the kidneys’ ability to concentrate urine, while albumin reflects proteinuria, a key symptom of CKD. Hemoglobin and packed cell volume are closely linked to anemia, a common complication of CKD caused by reduced erythropoietin production. Blood glucose random and hypertension represent the two leading causes of CKD worldwide, reflecting the model’s ability to capture key risk factors. Red blood cell count is a critical indicator, as CKD can impair erythropoiesis, leading to lower red blood cell production. For the alignment with clinical knowledge, specific gravity and albumin are integral to CKD diagnosis and staging. The inclusion of blood glucose random and hypertension reinforces the model’s clinical validity by recognizing the most common causes of CKD. Features such as hemoglobin and packed cell volume are vital for managing CKD complications like anemia, improving both patient outcomes and quality of life. For the practical Implications for clinicians, early detection of abnormalities in specific gravity and albumin can prompt timely interventions to prevent CKD progression Monitoring anemia related features such as hemoglobin and packed cell volume can help inform therapeutic strategies, including the use of erythropoiesis-stimulating agents or iron supplements. The model’s identification of hypertension and blood glucose random underlines the importance of managing these risk factors to mitigate CKD development. For the enhanced SHAP plot interpretation, positive SHAP values indicate features that increase the likelihood of CKD, while negative values reduce it. For instance, low specific gravity and high albumin levels are strong indicators of CKD, consistent with impaired kidney function. Similarly, low hemoglobin levels significantly drive CKD predictions, highlighting the established link between CKD and anemia.

### Statistical analysis results

Table 8 demonstrates the descriptive analysis for six models: XAI-CKD, RF, DT, BC, AdaBoost, and KNN. XAI-CKD model has the highest mean value at 80.12, that indicates better performance compared to other models. KNN model achieves the lowest mean value at 53.4546. The highest standard deviation is 44.2294 for the XAI-CKD model, this demonstrates that there is more variation in the model’s performance, the smallest standard deviation is 29.505854 for KNN model suggests more consistency in the results. For XAI-CKD model, the maximum value is 99.9, this indicates a good performance for the model. The 95% confidence interval for XAI-CKD is (25.2019, 135.0381), which indicates that the true mean of the model performance lies within the range. The 95% confidence interval for KNN is much smaller (16.8182, 90.0910), which means less uncertainty about the model performance. Table 9 presents the Wilcoxon rank sum test results of the proposed XAI-CKD model against several models. From the data demonstrated in this Table 9, the proposed XAI-CKD model outperforms the other models. Table 10 presents the ANOVA test results for the proposed XAI-CKD model for CKD prediction. The ANOVA table demonstrates a significant difference in performance, as indicated by the high F-statistic (76.72) and a very small P value (1.15 × 10⁻¹³).

**Table 8:** Descriptive analysis results of the proposed XAI-CKD model and another models.

Model	Mean	Standard deviation	Maximum	95% Confidence interval
XAI-CKD	80.12	44.229425	99.9	(25.2019, 135.0381)
RF	78.1736	43.159264	97.5	(24.5843, 131.7629)
DT	76.831	42.415973	95.8	(24.1646, 129.4974)
BC	75.4872	41.675410	94.2	(23.7403, 127.2341)
AdaBoost	68.2724	37.683783	85.3	(21.4818, 115.0630)
KNN	53.4546	29.505854	66.7	(16.8182, 90.0910)

**Table 9:** Wilcoxon rank sum test results of the proposed XAI-CKD model and another models.

Model	XAI-CKD	RF	DT	BC	AdaBoost	KNN
Theoretical Median	0.0	0.0	0.0	0.0	0.0	0.0
Actual Median	99.9	97.5	95.8	94.1	85.0	66.6
Number of Values	6	6	6	6	6	6
Sum of Signed Ranks (W)	0.0	0.0	0.0	0.0	0.0	0.0
Sum of Positive Ranks	0.0	0.0	0.0	0.0	0.0	0.0
Sum of Negative Ranks	0.0	0.0	0.0	0.0	0.0	0.0
P Value (Two-Tailed)	0.03125	0.03125	0.03125	0.03125	0.03125	0.03125
Exact or Estimate?	Exact	Exact	Exact	Exact	Exact	Exact
P Value Summary	**	**	**	**	**	**
Significant (alpha = 0.05)?	Yes	Yes	Yes	Yes	Yes	Yes
Discrepancy	99.9	97.5	95.8	94.1	85.0	66.6

**Table 10:** The ANOVA test results for the proposed XAI-CKD model for CKD prediction.

	SS	DF	MS	F (DFn, DFd)	*P* value
Treatment (between columns)	37975.402551	4	9493.850638	76.719301	1.15 × 10⁻¹³
Residual (within columns)	3093.696912	25	123.747876		
Total	41069.099463	29			

## Limitation of the work

The dataset used in this study consists of only 400 samples, which may leads to a potential risk of overfitting, especially when working with complex machine learning models like Extra Trees. The dataset used in this paper originates from a single region, which may restrict the model’s applicability to other populations. Factors such as demographics, geography, and socioeconomic conditions can significantly influence CKD prevalence and risk factors. The BBFS algorithm was employed to identify the most important features for CKD classification. However, we understand that feature selection might be influenced by the characteristics of the dataset used. This could lead to the exclusion of certain clinically relevant features. Although the model shows strong performance, its integration into clinical workflows may present practical and ethical challenges. For instance, clinicians may require additional tools to interpret and trust the model’s predictions before adopting them in their decision-making processes.

## Conclusion and future work

Chronic kidney disease (CKD) is a persistent condition characterized by the gradual deterioration of kidney function, typically assessed through the estimated glomerular filtration rate (eGFR) and the presence of kidney damage. The kidney disease improving global outcomes (KDIGO) organization has established a widely accepted system for categorizing CKD based on these factors. However, traditional machine learning models used for classification often lack transparency, making it difficult to understand the reasoning behind their decisions. To address this issue, we introduce the explainable artificial intelligence-CKD (XAI-CKD) model. This approach aims not only to accurately predict outcomes but also to provide clear and interpretable explanations for its decisions. XAI-CKD utilizes extra trees (ET) and shapley additive explanations (SHAP) values, along with the binary breadth-first search (BBFS) algorithm to select the most important features. By doing so, XAI-CKD seeks to offer valuable insights for improving decision-making in CKD classification. We compare the performance of XAI-CKD with several other machine learning models, including random forest (RF), decision tree (DT), bagging classifier (BC), adaptive boosting (AdaBoost), and k-nearest neighbor (KNN). Performance evaluation metrics such as accuracy, sensitivity, specificity, F-score, and area under the ROC curve (AUC) are utilized. Experimental results demonstrate that the proposed XAI-CKD model achieves the highest accuracy of 99.9%, showcasing its effectiveness in classifying CKD while providing transparent and understandable explanations for its decisions. In future work, we can collaborate with healthcare providers to validate the model’s performance in real-world clinical settings and integrating it into clinical workflows could facilitate its adoption for aiding healthcare professionals in CKD diagnosis and management, also expanding the XAI framework to other chronic diseases beyond CKD, such as diabetes or cardiovascular diseases, could broaden its applicability and impact in healthcare decision-making. Also, in the future LIME could provide localized insights into individual predictions, offering clinicians a clearer understanding of why specific decisions was made for each patient. By using attention mechanisms in deep learning models, we could highlight the most influential features in each prediction, creating intuitive visualizations that enhance transparency and trust. We also see significant potential in expanding the dataset to include other types of clinical information, such as: genetic information, longitudinal data. By combining these advanced interpretability techniques and additional data sources, future work could address current limitations and better capture the complexities of CKD.

## Ethical considerations in AI-Based CKD classification

Using patient data in AI models raises critical privacy concerns. Saving health information is essential. To address these concerns, we propose using data anonymization techniques to protect patient identities, ensuring secure storage and processing of data in controlled environments, and designing models with privacy to prevent unauthorized access or data misuse. AI models can sometimes reflect biases present in the training data, which could result in unequal outcomes for different demographic groups. This is particularly relevant for CKD classification, as prevalence and risk factors can vary significantly across populations. To address these concerns, we recommend implementing fairness aware algorithms to minimize bias in predictions. Also, we recognize the potential risks of model misuse, where predictions might be applied incorrectly or out of context, leading to unintended consequences. To prevent these concerns, we emphasize that the model should be used as a decision-support tool, not a standalone diagnostic system, training programs for clinicians should focus on interpreting AI outputs in the context of broader clinical information, monitoring and auditing mechanisms should be implemented to oversee the model’s deployment and ensure it is used appropriately. The focus on explainability in this study aligns with ethical principles of transparency. By making the model’s predictions interpretable through techniques like SHAP, we aim to help clinicians and patients understand the rationale behind the predictions. Additionally, we suggest incorporating feedback loops into future versions of the model to continuously evaluate and improve its ethical performance.

## Electronic supplementary material

Below is the link to the electronic supplementary material.

Supplementary Material 1

## Data Availability

https://www.kaggle.com/datasets/mansoordaku/ckdisease/data
