# Supplementary material for: Improved CKD classification based on explainable artificial intelligence with extra trees and BBFS
Source: Sci Rep. 2025 May 22;15:17861. doi: 10.1038/s41598-025-02355-7 (PMC12098988; doi:10.1038/s41598-025-02355-7)
Supplement: Supplementary file 1 — Supplementary Material 1 [file 41598_2025_2355_MOESM1_ESM.docx]

# **Appendix A**

# **Appendix A: Table of mathematical symbols.**

| **Symbol** | **Definition** |
| --- | --- |
| $x$ | Input feature |
| $\mu$ | Mean of the feature values |
| $\sigma$ | Standard deviation of the feature values |
| $z$ | $z$-score normalized value |
| TP | True positives |
| TN | True negatives |
| FP | False positives |
| FN | False negatives |
| Q | Queue used in BBFS algorithm for feature subset traversal |
| V | Number of vertices (features) in the feature selection graph |
| E | Number of edges in the BBFS search graph |
| S | Subset of selected features during BBFS traversal |
| S' | New subset formed by adding a feature to current subset S |

**Appendix B**

To test the performance of the proposed XAI-CKD model, we applied our proposed model on a public dataset available at https://www.kaggle.com/datasets/rabieelkharoua/chronic-kidney-disease-dataset-analysis. The dataset consists of 1659 instances and 54 features. Table 11 demonstrates the performance of the proposed XAI-CKD model compared to another five traditional machine learning models, namely, Random Forest (RF), Decision Tree (DT), Bagging Classifier (BC), Adaptive Boosting (AdaBoost), and K-Nearest Neighbor (KNN) in terms of accuracy, sensitivity, specificity, F-score, and Area Under the ROC Curve (AUC).

**Table 11.** Performance of the proposed XAI-CKD model and another traditional machine learning models.

| **Models** | **Accuracy** | **Sensitivity** | **Specificity** | **F-score** | **AUC** |
| --- | --- | --- | --- | --- | --- |
| **XAI-CKD** | **98.56%** | **98.57%** | **98.56%** | **98.57%** | **0.987** |
| **RF** | 95.63% | 95.63% | 95.64% | 95.63% | 0.959 |
| **DT** | 94.49% | 94.48% | 94.48% | 94.48% | 0.946 |
| **BC** | 92.81% | 92.81% | 92.82% | 92.81% | 0.929 |
| **AdaBoost** | 88.37% | 88.37% | 88.38% | 88.37% | 0.886 |
| **KNN** | 83.74% | 83.74% | 83.75% | 83.74% | 0.838 |

As depicted in Table 11, the proposed XAI-CKD model achieved the best results with accuracy of 98.56%, sensitivity of 98.57%, specificity of 98.56%, F-score of 98.57% that reflects a balance between precision and recall, and AUC of 0.987 that indicates excellent discrimination ability. The KNN model achieved the worth results with accuracy of 83.74%, sensitivity of 83.74%, specificity of 83.75%, F-score of 83.74% and AUC of 0.838. The second model achieved the best results after the proposed XAI-CKD model is RF model, its accuracy, sensitivity, specificity, F-score, and AUC are 95.63%, 95.63%, 95.64%, 95.63%, and 0.959. The third model achieved the best results after the RF model is DT model, its accuracy, sensitivity, specificity, F-score, and AUC are 94.49%, 94.48%, 94.48%, 94.48%, and 0.946. The fourth model achieved the best results after the DT model is BC model, its accuracy, sensitivity, specificity, F-score, and AUC are 92.81%, 92.81%, 92.82%, 92.81%, and 0.929. The fifth model achieved the best results after the BC model is Adaboost model, its accuracy, sensitivity, specificity, F-score, and AUC are 88.37%, 88.37%, 88.38%, 88.37%, and 0.886.
